# The Efficacy and Safety of Roxadustat for Anemia in Hemodialysis Patients with Chronic Kidney Disease: A Meta-Analysis of Randomized Controlled Trials

**DOI:** 10.3390/toxics12120846

**Published:** 2024-11-25

**Authors:** Yunling Geng, Shuaixing Zhang, Zijing Cao, Jingyi Tang, Hailan Cui, Zhaocheng Dong, Yuning Liu, Weijing Liu

**Affiliations:** 1 Key Laboratory of Chinese Internal Medicine of Ministry of Education and Beijing, Renal Research Institution of Beijing University of Chinese Medicine, Dongzhimen Hospital Affiliated to Beijing University of Chinese Medicine, Beijing 100700, China; 20210941130@bucm.edu.cn (Y.G.); 20210941131@bucm.edu.cn (S.Z.); caozj2021@126.com (Z.C.); tangjingyi1997@163.com (J.T.); dzc19950502@163.com (Z.D.); 2 Beijing Changping Hospital of Traditional Chinese Medicine, Beijing 102200, China; yilisha1019@126.com

**Keywords:** roxadustat, anemia, hemodialysis, chronic kidney disease, meta-analysis

## Abstract

Background: Patients undergoing hemodialysis (HD) for chronic kidney disease (CKD) often encounter anemia. Roxadustat has not only undergone phase II-III clinical trials in patients suffering from CKD and undergoing HD; a number of post-marketing clinical studies have been conducted using the drug. This article was to assess the effectiveness and safety of roxadustat in managing anemia among patients with CKD undergoing HD. Methods: A thorough search was performed across eight databases, including PubMed, Web of Science, Cochrane Library, Embase, Wan Fang, China National Knowledge Infrastructure (CNKI), Chongqing VIP (CQ VIP), and SinoMed to identify randomized clinical trials (RCTs) examining the effectiveness and safety of roxadustat in managing anemia among individuals suffering from CKD and undergoing HD. This search included studies from the inception of these databases to April 2023. Results: Two phase II, one phase III, and 16 post-marketing studies with 1688 participants were included. Serum iron (SI), transferrin, and total iron-binding capacity (TIBC) levels changed from baseline (∆SI, ∆transferrin, and ∆TIBC) and were significantly more increased for roxadustat than for erythropoiesis-stimulating agents (ESAs): MD 2.55, (95% CI 1.51 to 3.60), *p* < 0.00001; MD 0.55, (95% CI 0.41 to 0.69), *p* < 0.00001; and MD 6.54, (95% CI 4.50 to 8.59), *p* < 0.00001, respectively. Roxadustat was not inferior to ESAs with regard to increasing Hb (∆Hb) levels [MD 1.17 (95% CI 0.71 to 1.63), *p* < 0.00001] (g/dL). No statistically significant distinctions of the ∆ferritin, ∆hepcidin, and transferrin saturation (TSAT) from baseline (∆TSAT) level were identified between roxadustat and ESAs. C-reactive protein (CRP) levels changed from baseline (∆CRP) and were significantly more reduced for roxadustat than for ESAs. As for safety, the analysis indicated no notable difference in the occurrence of adverse events (AEs) and serious adverse events (SAEs) between roxadustat and ESAs. Conclusions: This meta-analysis demonstrated that roxadustat outperformed ESAs in enhancing SI, transferrin, and TIBC levels while also decreasing CRP levels. Roxadustat was not inferior to ESAs in terms of improving Hb levels and safety. These findings suggest that roxadustat was well tolerated and a potent alternative to ESAs in managing anemia among patients suffering from CKD and undergoing HD.

## 1. Introduction

Chronic kidney disease (CKD) constitutes a serious global health concern, characterized by diminished kidney function or elevated urinary protein, and it affects 15–20% of adults worldwide [1]. The Kidney Disease Improving Global Outcomes (KDIGO)’s recommendations stipulate that a diagnosis of chronic kidney disease (CKD) necessitates an estimated glomerular filtration rate (eGFR) below 60 mL/min/1.73 m^2^ sustained for 3 consecutive months or more, or the presence of significant albuminuria. Anemia frequently occurs in hemodialysis (HD) patients with CKD, stemming from reduced erythropoietin production linked to compromised kidney function and disrupted iron metabolism, leading to lowered standard of life and heightened illness and death [2]. Anemia occurs in 15.4% of individuals with CKD, nearly double the prevalence of 7.6% found in the general population. The data originate from cross-sectional analyses of the National Health and Nutrition Examination Survey (NHANES) conducted in the years 2007–2008 and 2009–2010 [3]. Anemia prevalence escalates with the advancement of CKD, reaching rates of up to 53.4% in stage 5 CKD. Erythropoiesis-stimulating agents (ESAs), in conjunction with iron supplementation, are utilized in the treatment of anemia as per current Clinical Practice Guidelines [4,5].

Although research on the early application of ESAs has validated their efficacy in alleviating anemia symptoms, diminishing the necessity for blood transfusions, and enhancing quality of life, the safety of elevated ESA dosages and hemoglobin concentration targets during ESA treatment remains contentious [6]. A countrywide cohort study conducted among patients in Japan undergoing HD revealed that individuals administered long-acting ESAs exhibited a 13% increased mortality rate compared to those who were administered short-acting agents, following a two-year follow-up period [7]. Nonetheless, another study indicated that elevated doses of ESAs may correlate with a heightened risk of advancing to ESRD or mortality in comparison to long-acting ESAs [8]. The Correction of Hemoglobin Outcomes in Renal Insufficiency (CHOIR) trial demonstrated that aiming for a target Hb concentration of 13.5 g/dL was related to an elevated combined risk of death, myocardial infarction, stroke, and hospitalization due to congestive heart failure while not yielding any enhancement in life quality [9]. Furthermore, the efficacy of ESAs is decreased in patients under inflammatory conditions [10]. Likewise, intravenous (IV) iron therapy presents several restrictions, including the discomfort of injections and potential hazards such as heightened infection risk, headaches, hypotension, and hypersensitivity reactions [11]. Oral iron formulations, except for the phosphate binder ferric citrate, seem to lack efficacy in hemodialysis patients [12]. In addition, the tolerance and compliance with oral iron preparations might be reduced due to gastrointestinal intolerance and constipation [13]. Consequently, alternate therapy for patients with CKD undergoing HD, particularly oral pharmaceutical alternatives, is presently under investigation.

Hypoxia-inducible factor (HIF) functions as a transcription factor that governs erythropoiesis and is degraded by prolyl hydroxylase enzymes in natural oxygen environments. Under hypoxic conditions, prolyl hydroxylase enzyme activity is suppressed, facilitating the interaction between HIF-α and HIF-β. This interaction brings about enhanced erythropoiesis, higher iron absorption, and reduced hepcidin levels [14]. It has been reported that HIF prolyl hydroxylase inhibitors (HIF-PHIs) may enhance the expression of EPO and regulate iron metabolism [14]. Roxadustat received approval in China in December 2018, in Japan by September 2019, and in Europe by August 2021 for treating CKD anemia and is presently undergoing investigation in the United States [15]. Currently, there is one phase III study and two phase II studies, along with other post-marketing studies, that are assessing the application of roxadustat among patients with CKD receiving HD. Despite prior meta-analyses indicating that roxadustat could improve Hb levels and is typically tolerated fairly well in DD-CKD patients, the proof among individuals undergoing HD with CKD remains inadequate [16,17]. Additionally, a study assessing the effectiveness as well as safety of roxadustat in patients receiving HD included only Chinese individuals, hence constraining the applicability of the results to diverse ethnic groups [18]. This article sought to evaluate the effectiveness as well as safety of roxadustat in managing anemia among individuals suffering from CKD and undergoing HD, thereby offering more robust evidence for its administration.

## 2. Materials and Methods

The meta-analysis adhered to the Methodological Expectations of Cochrane Intervention Reviews (MECIR) guidelines, and its findings were reported in alignment with the Preferred Reporting Items for Systematic Reviews and Meta-Analyses (PRISMA) Statement. Furthermore, this research has been recorded in the International Prospective Register of Systematic Reviews (PROSPERO) database (number CRD42023428434).

### 2.1. Search Strategy

A thorough search was performed across 8 databases, including PubMed, Web of Science, Cochrane Library, Embase, Wan Fang, China National Knowledge Infrastructure (CNKI), Chongqing VIP (CQ VIP), and SinoMed, to identify randomized clinical trials (RCTs) investigating the effectiveness as well as safety of roxadustat in managing anemia among patients suffering from CKD and undergoing HD. This search encompassed the literature from the inception of these databases up to April 2023. Additionally, relevant published meta-analyses were also reviewed. The specific retrieval strategies employed in this search are detailed in the Supplementary Material.

### 2.2. Inclusion and Exclusion Criteria

Trials meeting the specified criteria were selected:

(1) RCTs investigating the effectiveness and safety of roxadustat in managing anemia. (2) Patients diagnosed with CKD and receiving maintenance HD, without race, language, gender, or age restrictions. (3) The experimental cohort received roxadustat, while the control group underwent therapy with ESAs. (4) The primary outcome was the change in hemoglobin (Hb) levels from baseline. Secondary outcomes assessed variations in serum iron (SI), hepcidin, ferritin, transferrin, total iron-binding capacity (TIBC), and transferrin saturation (TSAT). Adverse events (AEs) and serious adverse events (SAEs) were documented to evaluate roxadustat’s safety profile.

Studies that met any of the exclusion standards were excluded:

(1) Non-RCTs, including animal studies, retrospective studies, case reports, and reviews. (2) Incomplete baseline information of patients. (3) Failure to report the changes in Hb levels in the data.

### 2.3. Study Selection and Data Extraction

Following the elimination of duplicate studies, two researchers (C.Z.J. and T.J.Y.) independently selected and extracted data from the eligible studies that met the eligibility criteria by reviewing the titles, abstracts, and entire papers. A third reviewer (C.H.L.) was involved in making decisions when there were disagreements on study selection and data extraction. The information retrieved encompassed the characteristics of the study (publication year, name of first author, country, single or multicenter designation, intervention, control, and duration), patient characteristics (sample size, gender ratio, age, and baseline Hb levels), and outcomes (primary and secondary outcomes).

### 2.4. Assessment of Bias Risk and Evidence Quality

Two researchers (T.J.Y. and C.Z.J.) conducted independent assessments of bias risk and evidence quality. The bias risk was evaluated via the Collaboration’s tool. Items were categorized into three levels: low risk, high risk, and unclear risk. The evidence quality was assessed employing the Grading of Recommendation, Assessment, Development, and Evaluation (GRADE) method. The evidence quality was assessed as high, moderate, low, or very low based on five items, including study limitations, inconsistency, indirectness, imprecision, and other factors. A third researcher (C.H.L.) was involved in resolving the disagreement.

### 2.5. Statistical Analysis

The outcome data were analyzed using Cochrane’s tool. After that, the meta-analysis was performed using RevMan 5.3 and Stata 14. Continuous data results were reported as the mean difference (MD) or standard mean difference (SMD) accompanied by a 95% confidence interval (CI) between the roxadustat and ESA groups. Dichotomous outcomes were investigated using the risk ratio (RR) with a corresponding 95% CI. The heterogeneity was calculated using I^2^ and *p* values. A random-effects model was adopted to show the findings for all outcomes.

### 2.6. Subgroup and Sensitivity Analyses

If I^2^ ≥ 50%, subgroup and sensitivity analyses were performed to find the source of heterogeneity. The subgroup analysis was conducted based on the phases of the clinical trials. Sensitivity analysis was conducted by sequentially removing each trial to evaluate whether a single trial could influence the heterogeneity and pooled results.

### 2.7. Publication Bias Analysis

Egger’s test and funnel plot analyses were conducted to evaluate the publication bias concerning both primary and secondary outcomes.

## 3. Results

### 3.1. Study Characteristics

**Figure 1:**
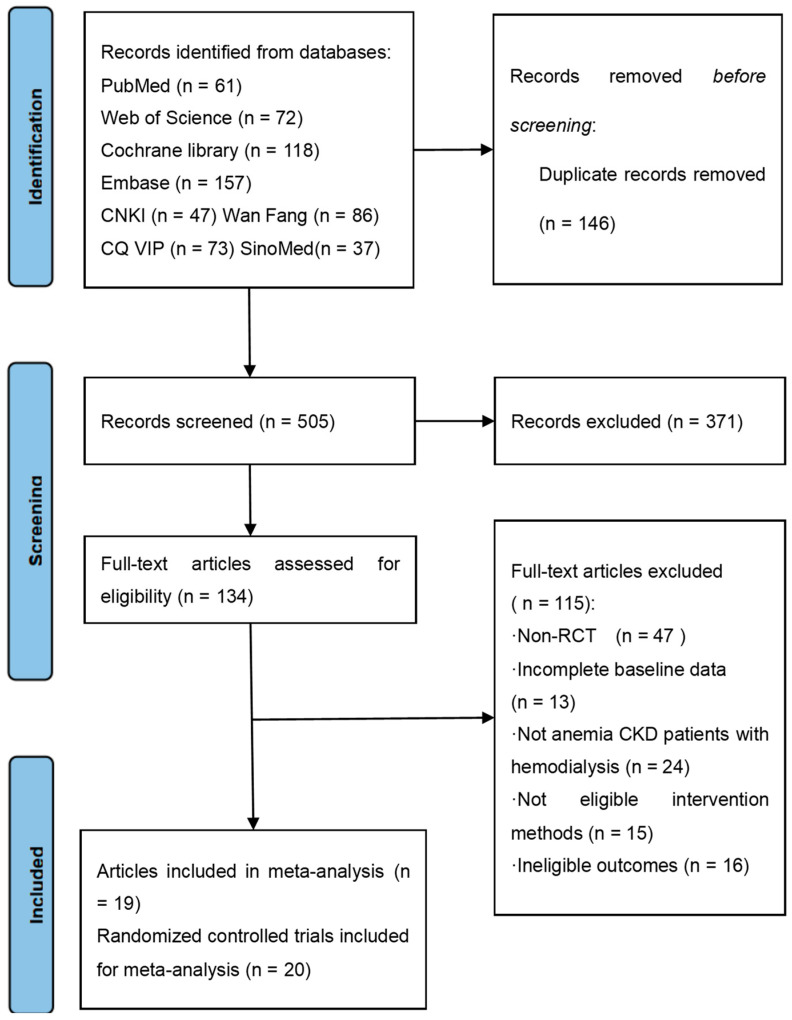
Flowchart of this study.

An aggregate of 651 articles were recognized according to the retrieval strategy, of which 146 duplicate publications were removed. Among the remaining 505 articles, 371 were excluded after reviewing the titles as well as the abstracts, while 134 articles were selected for eligibility assessment based on full texts. Subsequently, 115 articles were ruled out for the subsequent reasons: non-RCTs (*n* = 47), incomplete baseline data (*n* = 13), not including CKD anemic patients on hemodialysis (*n* = 24), not eligible intervention methods (*n* = 15), and ineligible outcomes (*n* = 16). Finally, 19 articles with 20 trials were included for meta-analysis [19] (Figure 1).

We studied 20 RCTs in which 1688 patients were enrolled [20,21,22,23,24,25,26,27,28,29,30,31,32,33,34,35,36,37,38]. Among them, 1 study was performed in Japan [20], 2 trials in the United States [21], and the remaining 17 RCTs in China. The primary features of the studies are shown in Table 1.

### 3.2. Evaluation of the Risk of Bias

**Figure 2:**
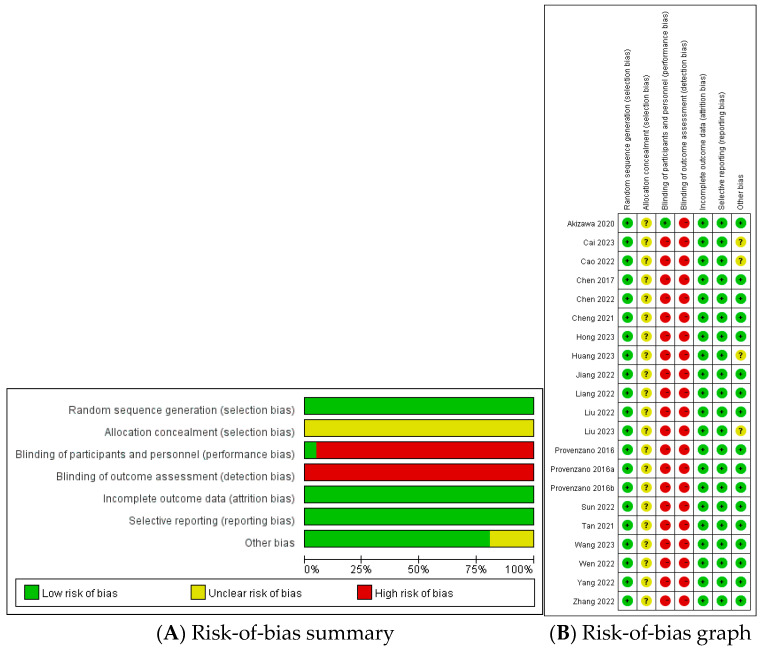
Risk-of-bias assessment using the Cochrane Risk-of-Bias tool. Various colors and symbols denote distinct levels of bias risk; specifically, green and “+” represent a low risk, yellow and “?” signify an unclear risk, red and “−” denote a high risk. Bars represent the overall bias risk associated with each item. Circles represent the bias risk of each item corresponding to each study.

The bias risk was evaluated based on the Cochrane Collaboration’s Risk-of-Bias tool. All studies had an unclear risk of bias regarding allocation concealment and a high risk of bias concerning blinding of outcome data, considering that medication dose should be adjusted to maintain the level of Hb. Only one RCT used a double-blind, double-dummy method to conduct the trial [20]. All RCTs had low risk of selective reporting and incomplete data. However, four RCTs had another unclear bias: one did not report whether iron supplements were used [28], one did not mention prior use of ESAs or roxadustat [23], and two did not report AEs [22,31]. The evaluation results are presented in Figure 2.

### 3.3. Meta-Analysis

#### 3.3.1. Primary Outcome

##### Changes in Hb Level from Baseline (∆Hb)

**Figure 3:**
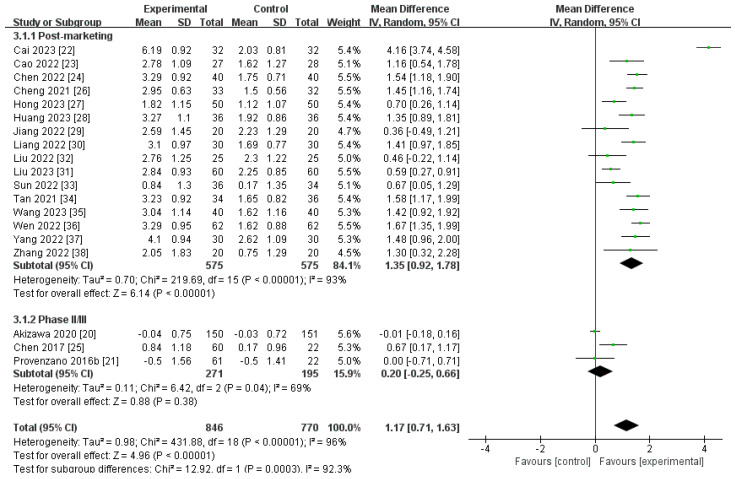
The forest graph of ∆Hb (g/dL). The green square represents the point estimate of the effect size of each study, the line segment length represents the 95% CI of the effect size of each study, the diamond represents the summary results of the meta-analysis synthesis of each study, and the diamond width represents the 95% CI of the effect size of the summary results.

A total of 19 studies, containing 1616 participants, compared the ∆Hb level of roxadustat with ESAs [20,21,22,23,24,25,26,27,28,29,30,31,32,33,34,35,36,37,38]. A notable level of heterogeneity was observed (I^2^ = 96%, *p* < 0.00001), prompting the application of a random-effects model. The aggregated findings demonstrated that the effectiveness of increasing hemoglobin (∆Hb) levels was comparable to that observed in the control group [MD 1.17 (95% CI 0.71 to 1.63), *p* < 0.00001] (g/dL) (Figure 3; Table 2). Similarly, at the end of treatment, the Hb level was increased and remained similar for roxadustat and ESAs [MD 1.15 (95% CI 0.75 to 1.55), *p* < 0.00001] (g/dL) (Figure S1). In the post-marketing subgroup, the results indicated that the ∆Hb levels were found to be greatly elevated in the roxadustat cohort compared to the cohort receiving ESAs [MD 1.35 (95% CI 0.92 to 1.78), *p* < 0.00001] (g/dL). However, in the phase II or III subgroup, no noteworthy distinction was observed between the roxadustat and ESA groups. Sensitivity analyses showed that there was no apparent change in the statistical results, despite no heterogeneity in the phase II or III subgroup after ruling out the study conducted by Chen [25] (Figure S2).

#### 3.3.2. Secondary Outcomes

##### Changes in SI Levels from Baseline (∆SI)

**Figure 4:**
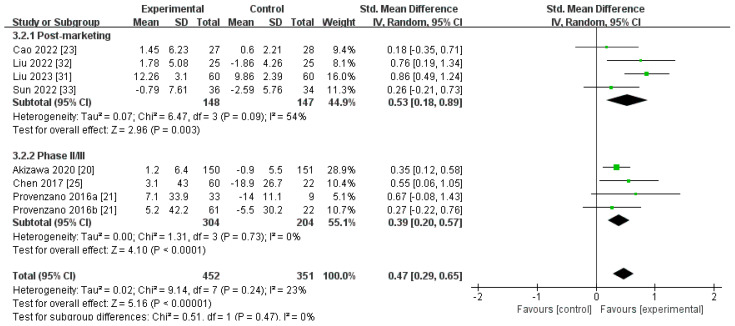
The forest graph of ∆SI (umol/L, ug/dL, mmol/L, or ug/mL). The green square represents the point estimate of the effect size of each study, the line segment length represents the 95% CI of the effect size of each study, the diamond represents the summary results of the meta-analysis synthesis of each study, and the diamond width represents the 95% CI of the effect size of the summary results.

Eight RCTs, containing 803 participants, compared the ∆SI level for roxadustat versus ESAs [20,21,23,25,31,32,33]. The aggregated findings derived from a random-effects model indicated that the ∆SI level was markedly higher for roxadustat than for ESAs [SMD 0.53, (95% CI 0.18 to 0.89), *p* = 0.003; SMD 0.39, (95% CI 0.20 to 0.57), *p* < 0.0001, respectively; and SMD 0.47, (95% CI 0.29 to 0.65), *p* < 0.00001, combined] (Figure 4; Table 2). After harmonization of units (umol/L), the statistical results revealed that the ∆SI level was more elevated in individuals treated with roxadustat than those receiving ESAs [MD 2.50, (95% CI 1.61 to 3.39), *p* < 0.00001; MD 2.62, (95% CI 0.05 to 5.18), *p* = 0.05, respectively; and MD 2.55, (95% CI 1.51 to 3.60), *p* < 0.00001, combined] (Figure S3). Sensitivity analyses showed that there was no significant heterogeneity after excluding the study carried out by Liu in 2023 [31] (Figure S4).

##### Changes in Hepcidin Levels from Baseline (∆Hepcidin)

**Figure 5:**
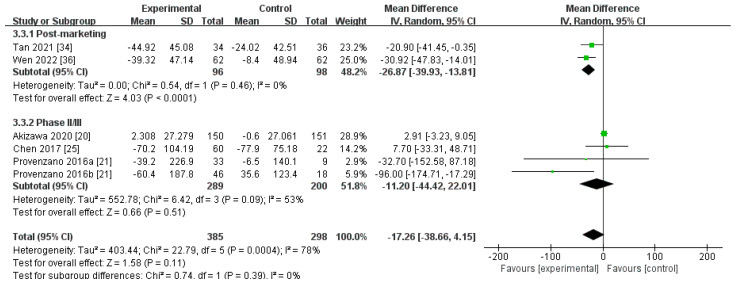
The forest graph of ∆hepcidin (ng/mL). The green square represents the point estimate of the effect size of each study, the line segment length represents the 95% CI of the effect size of each study, the diamond represents the summary results of the meta-analysis synthesis of each study, and the diamond width represents the 95% CI of the effect size of the summary results.

Six RCTs, involving 683 participants, compared the ∆hepcidin level between the roxadustat cohort and the ESA cohort [20,21,25,34,36]. The aggregated findings utilizing a random-effects model indicated that no statistically noteworthy variations were observed in the ∆hepcidin level between roxadustat and ESAs [MD −17.26, (95% CI −38.66 to 4.15), *p* = 0.11] (Figure 5; Table 2). However, in the post-marketing subgroup, the results demonstrated that the ∆hepcidin level was markedly reduced for roxadustat when compared to ESAs [MD −26.87, (95% CI −39.93 to −13.81), *p* < 0.0001]. After eliminating the study performed by Akizawa [20], the pooled sensitivity analysis of the two subgroups showed that the ∆hepcidin level was considerably reduced in the roxadustat cohort compared to the ESA cohort (I^2^ = 35%, *p* = 0.18), [MD −24.74, (95% CI −43.39 to −6.09), *p* = 0.009] (Figure S5).

##### Changes in Ferritin Levels from Baseline (∆Ferritin)

**Figure 6:**
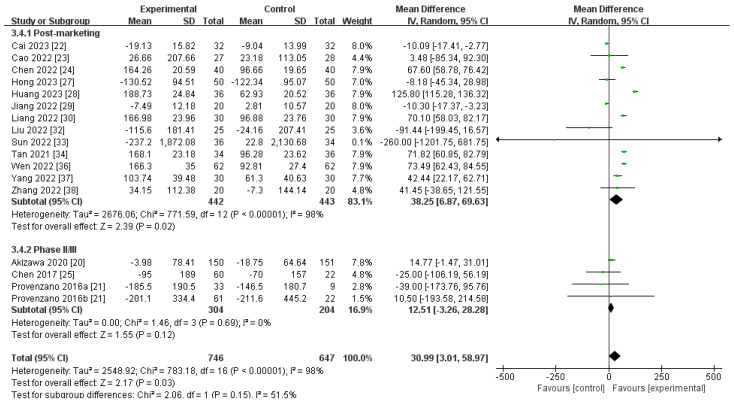
The forest graph of ∆ferritin (ng/mL). The green square represents the point estimate of the effect size of each study, the line segment length represents the 95% CI of the effect size of each study, the diamond represents the summary results of the meta-analysis synthesis of each study, and the diamond width represents the 95% CI of the effect size of the summary results. The arrow indicates that the 95% CI for the study’s effect size is outside the graphical range, and the excess is indicated by the arrow.

A total of 17 RCTs, comprising 1393 participants, compared the ∆ferritin level in the cohort receiving roxadustat compared to the control cohort [20,21,22,23,24,25,27,28,29,30,32,33,34,36,37,38]. The aggregated findings utilizing a random-effects model showed that the ∆ferritin level was significantly higher in the roxadustat cohort than in the cohort receiving ESAs [MD 30.99, (95% CI 3.01 to 58.97), *p* = 0.03] (Figure 6; Table 2). However, in the phase II or III subgroup, no statistically noteworthy distinction in ∆ferritin levels was detected between roxadustat and ESAs. Sensitivity analyses showed that the heterogeneity was not significantly reduced by the sequential removal of a single trial.

##### Changes in Transferrin Levels from Baseline (∆Transferrin)

**Figure 7:**
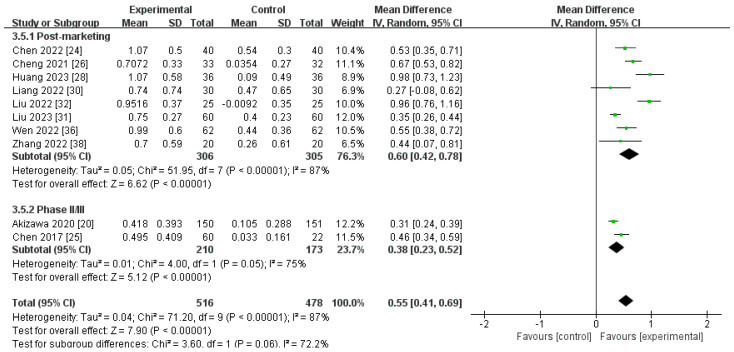
The forest graph of ∆transferrin (g/L). The green square represents the point estimate of the effect size of each study, the line segment length represents the 95% CI of the effect size of each study, the diamond represents the summary results of the meta-analysis synthesis of each study, and the diamond width represents the 95% CI of the effect size of the summary results.

A total of 10 trials, with 994 individuals, compared the ∆transferrin level for roxadustat versus for ESAs [20,24,25,26,28,30,31,32,36,38]. The heterogeneity test showed significant heterogeneity (I^2^ = 87%, *p* < 0.00001). The aggregated findings demonstrated that the ∆transferrin level was markedly more elevated for roxadustat than for ESAs [MD 0.60, (95% CI 0.42 to 0.78), *p* < 0.00001 and MD 0.38, (95% CI 0.23 to 0.52), *p* < 0.00001, respectively; and MD 0.55, (95% CI 0.41 to 0.69), *p* < 0.00001, combined] (Figure 7; Table 2). Sensitivity analysis showed that not a single trial could change the heterogeneity results.

##### Changes in TIBC Levels from Baseline (∆TIBC)

**Figure 8:**
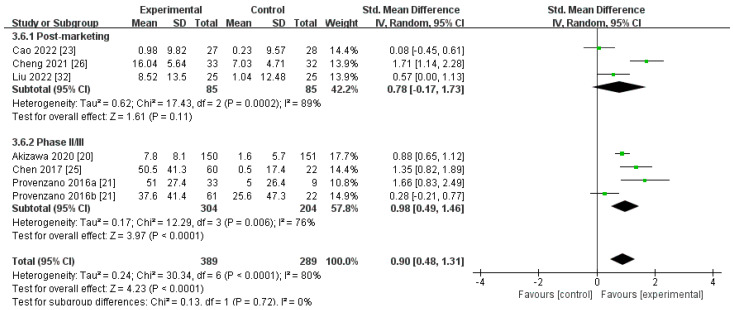
The forest graph of ∆TIBC (umol/L or ug/dL). The green square represents the point estimate of the effect size of each study, the line segment length represents the 95% CI of the effect size of each study, the diamond represents the summary results of the meta-analysis synthesis of each study, and the diamond width represents the 95% CI of the effect size of the summary results.

Seven trials, consisting of 678 participants, compared the ∆TIBC level for roxadustat versus ESAs [20,21,23,25,26,32]. The aggregated findings revealed that the ∆TIBC level was apparently higher for roxadustat than for ESAs [SMD 0.90, 95% CI 0.48 to 1.31, *p* < 0.0001] (Figure 8; Table 2). After harmonization of units (umol/L), the statistical results showed that the ∆TIBC level was more elevated for roxadustat than for ESAs [MD 5.92, (95% CI 0.41 to 11.42), *p* = 0.04; MD 6.65, (95% CI 4.27 to 9.04), *p* < 0.00001, respectively; and MD 6.54, (95% CI 4.50 to 8.59), *p* < 0.00001, combined] (Figure S6). Sensitivity analysis showed that there was no obvious change after removing a single study one by one.

##### Changes in TSAT Levels from Baseline (∆TSAT)

**Figure 9:**
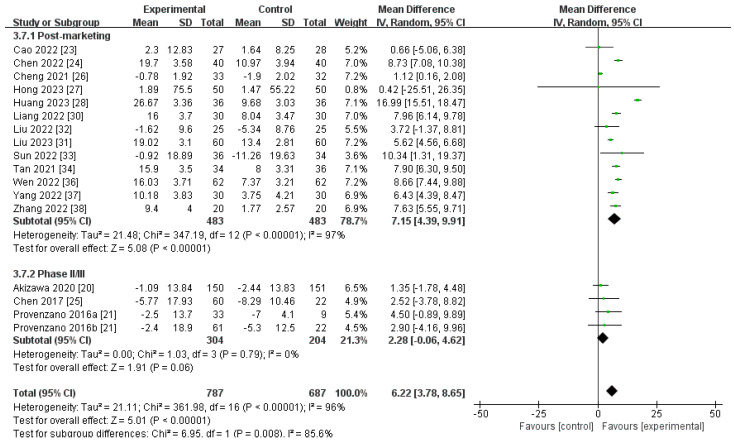
The forest graph of ∆TSAT (%). The green square represents the point estimate of the effect size of each study, the line segment length represents the 95% CI of the effect size of each study, the diamond represents the summary results of the meta-analysis synthesis of each study, and the diamond width represents the 95% CI of the effect size of the summary results.

A total of 17 trials, containing 1474 participants, compared the ∆TSAT level for roxadustat versus ESAs [20,21,23,24,25,26,27,28,30,31,32,33,34,36,37,38]. The aggregated findings indicated that the ∆TSAT level was significantly higher for roxadustat than for ESAs [MD 6.22, (95% CI 3.78 to 8.65), *p* < 0.00001]. Nevertheless, no notable distinction was observed in the ∆TSAT level between roxadustat and ESAs within the phase II or III subgroup (Figure 9; Table 2). Sensitivity analysis showed that not a single trial could change the result of the heterogeneity test.

##### Changes in CRP Levels from Baseline (∆CRP)

**Figure 10:**
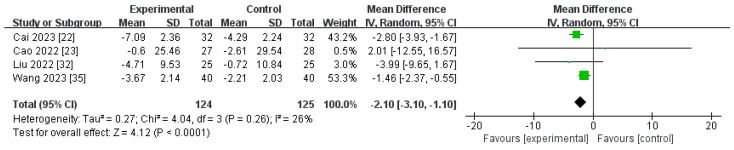
The forest graph of ∆CRP (mg/L). The green square represents the point estimate of the effect size of each study, the line segment length represents the 95% CI of the effect size of each study, the diamond represents the summary results of the meta-analysis synthesis of each study, and the diamond width represents the 95% CI of the effect size of the summary results.

Four RCTs, containing 249 individuals, compared the ∆CRP level for roxadustat versus ESAs [22,23,32,35]. The pooled results showed that the ∆CRP level was markedly reduced in individuals administered roxadustat compared to those receiving ESAs [MD −2.10, (95% CI −3.10 to −1.10), *p* < 0.0001] (Figure 10; Table 2).

### 3.4. The Safety

#### 3.4.1. AEs

**Figure 11:**
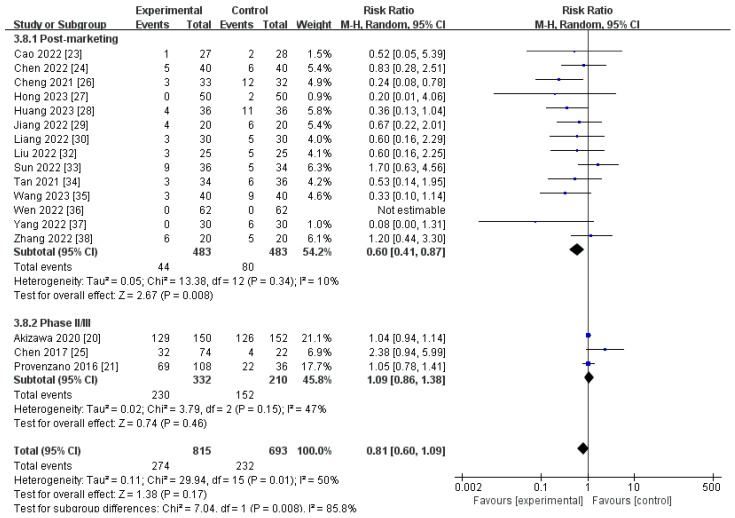
The forest graph of AEs. The blue square represents the point estimate of the effect size of each study, the line segment length represents the 95% CI of the effect size of each study, the diamond represents the summary results of the meta-analysis synthesis of each study, and the diamond width represents the 95% CI of the effect size of the summary results.

A total of 17 trials, including 1508 participants, mentioned the AEs for roxadustat versus ESAs [20,21,23,24,25,26,27,28,29,30,32,33,34,35,36,37,38]. AEs included adverse vital signs, such as temperature, pulse, respiration, and blood pressure, and uncomfortable clinical symptoms, such as dizziness, nausea, and diarrhea, as well as abnormal laboratory markers. AEs were coded to a standardized set of terms using the Adverse Event Dictionary from the Medical Dictionary for Regulatory Activities and recorded in detail in the clinical trial report form; they were not necessarily related to the use of medication. The meta-analysis revealed that there was no notable disparity in the occurrence of AEs between roxadustat and ESAs [RR 0.81, (95% CI 0.60 to 1.09), *p* = 0.17]. However, in the post-marketing subgroup, the incidence of AEs was significantly lower for roxadustat than for ESAs [RR 0.60, (95% CI 0.41 to 0.87), *p* = 0.008] (Figure 11; Table 2). Sensitivity analysis discovered that no individual study had the capacity to significantly influence the statistical results.

#### 3.4.2. SAEs

**Figure 12:**
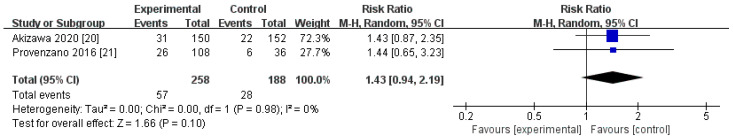
The forest graph of SAEs. The blue square represents the point estimate of the effect size of each study, the line segment length represents the 95% CI of the effect size of each study, the diamond represents the summary results of the meta-analysis synthesis of each study, and the diamond width represents the 95% CI of the effect size of the summary results.

Only two trials reported the incidence of SAEs in the treatment of roxadustat and ESAs [20,21]. Akizawa et al. [20] reported that 20.7% (31/150) of roxadustat-treated patients and 14.5% (22/152) of darbepoetin alfa-treated patients experienced at least one SAE. The SAEs consisted of cardiac disorders, gastrointestinal disorders, injury, poisoning and procedural complications, nervous system disorders, vascular disorders requiring hospitalization or resulting in death, and so on. Two cases of deep vein thrombosis were found in the roxadustat group. Provenzano et al. [21] reported that 26 of 108 (24.1%) participants for roxadustat and 6 of 36 (17%) participants for epoetin alfa were found to have experienced SAEs. Four cardiac disorders, including two acute myocardial infarctions, one instance of congestive cardiac failure, and one cardiorespiratory arrest, for the roxadustat group and two cardiac disorders, consisting of one instance of coronary artery disease, one cardiac arrest, one instance of congestive cardiac failure, and one myocardial infarction, for the epoetin alfa group were observed, respectively. The aggregated findings from the random-effects model revealed that the occurrence of SAEs was not statistically markedly different in both treatments [RR 1.43, (95% CI 0.94 to 2.19), *p* = 0.10] (Figure 12; Table 2).

### 3.5. Publication Bias

**Figure 13:**
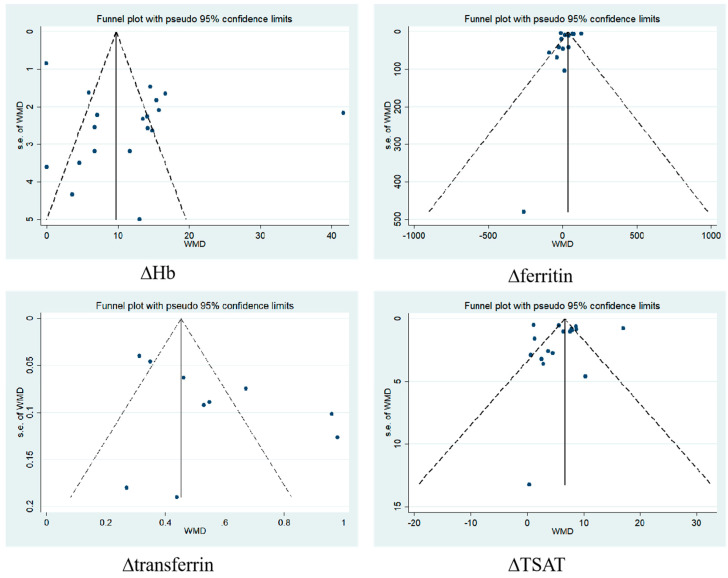
Publication bias analysis. The dots in the funnel plot represent the included studies, the horizontal axis represents the effect size, the vertical axis represents the s.e., the vertical line in the middle represents the ideal effect size, and the two diagonal lines represent the 95% CI.

Egger’s test and funnel plot analyses were used to evaluate publication bias (Table 3). Our meta-analysis indicated no publication bias in all results, and the funnel plots of ∆Hb, ∆ferritin, ∆transferrin, and ∆TSAT were as follows (Figure 13).

### 3.6. Quality of Evidence

The evidence quality for the efficacy and safety of roxadustat on all outcomes was rated as “very low” (Table 4).

## 4. Discussion

Our study contained current RCTs to evaluate the effectiveness as well as safety of roxadustat in managing anemia among individuals with CKD undergoing HD including both phase II or III clinical trial studies and post-marketing clinical trial studies. The aggregated findings indicated that roxadustat outperformed ESAs in enhancing SI, transferrin, and TIBC levels while also reducing CRP levels. Simultaneously, roxadustat showed comparability to ESAs in elevating Hb, ferritin, and TSAT levels and lowering hepcidin levels. The occurrence of AEs and SAEs did not exhibit notable differences between the roxadustat cohort and ESA cohort. The evidence quality for all outcome analyses was assessed as very low.

Roxadustat (also named FG-4592 or ASP1517), a first-in-class orally active HIF-PHI, reversibly stabilizes the HIF-α subunit and facilitates its dimerization with HIF-β, hence promoting erythropoiesis, iron uptake, mobilization, and transport irrespective of inflammation [39]. Several studies into the pharmacokinetic and pharmacodynamic characteristics of roxadustat among individuals with CKD undergoing HD have demonstrated that roxadustat can transiently elevate endogenous EPO levels, similar to those observed in healthy individuals under hypoxic circumstances, for instance at high altitudes (NCT02965040 in Germany and the United Kingdom; FGCL-4592-039 in the United States) [40,41]. It is reported that less than 5% of roxadustat and its metabolites are eliminated by HD, with around 99% binding to plasma proteins [41]. This results in a flexible drug delivery regimen, allowing roxadustat to be provided before or after dialysis without the worry of impaired efficacy. Furthermore, no major adverse events related to treatment or dosage were noted, including among patients with end-stage renal illness, indicating that roxadustat demonstrates a favorable tolerance profile across diverse populations, irrespective of the degree of renal function impairment [41]. These findings were congruent with earlier phase II or III outcomes. Currently, not only phase II or III RCTs but also several post-marketing RCTs have been conducted on roxadustat for managing anemia among individuals suffering from CKD undergoing HD. Thus, it is essential to evaluate the effectiveness and safety of roxadustat compared to ESAs in patients with CKD undergoing HD to furnish more credible proof for the clinical utilization of roxadustat.

The primary outcome of our meta-analysis indicated that roxadustat was equally as effective as ESAs in enhancing Hb levels, consistent with prior meta-analyses [42,43]. In the meta-analysis, roxadustat was comparable to ESAs in elevating Hb levels in the dialysis-dependent category. In the phase II or III subgroup, two randomized, open-label phase II studies sponsored by Astellas Pharma (NCT01596855, NCT01147666) and one randomized, double-blind, active comparator-controlled phase III study sponsored by FibroGen (NCT02952092) were conducted in CKD patients with anemia reliant on HD, indicating that roxadustat demonstrated comparable efficacy to epoetin alfa and was noninferior to darbepoetin alfa [20,21,25]. Despite the ∆Hb level being markedly elevated for roxadustat compared to ESAs in the post-marketing subgroup, we could not conclude that roxadustat was superior to ESAs in enhancing Hb levels due to considerable heterogeneity. Furthermore, the implications of the elevated Hb levels associated with roxadustat, in comparison to ESAs, necessitate further investigation to determine whether this elevation constitutes a benefit or a potential risk. Clinically, the extent of Hb increase is closely associated with improvements in life quality, alleviation of fatigue, and enhancement of daily functioning among patients. However, such increases in Hb may also elevate the risk of altered blood viscosity, which could subsequently result in an increased occurrence of cardiovascular events, including hypertension, thrombosis, and stroke. SAEs were documented in the phase II and III trials included in the analysis, yet these were not observed in the post-marketing studies. The question of whether roxadustat possesses unique pharmacological characteristics that mitigate the risk of surpassing recommended Hb levels remains to be substantiated through long-term post-marketing real-world studies (RWSs) or observational research. Consequently, additional investigations are warranted to comprehensively evaluate whether the elevation of Hb levels through roxadustat results in clinical benefits, alongside any associated risks.

The secondary outcomes of our meta-analysis indicated that roxadustat significantly elevated SI, transferrin, and TIBC levels in comparison to the ESA group. The aggregated results indicated no notable discrepancies in hepcidin, ferritin, and TSAT levels between roxadustat and ESAs. Iron, a principal constituent of hemoglobin, participates in numerous critical biological processes, such as energy metabolism, DNA synthesis, and the detoxification of reactive oxygen compounds, all of which are essential for sustaining life. In patients with CKD, particularly those undergoing HD, iron shortage, manifesting as both absolute and functional deficiencies of iron, frequently occurs in the context of chronic inflammation [44]. Recently, iron shortage has been addressed with IV iron supplementation, which temporarily elevates iron levels without enhancing the overall availability of iron, necessitating repeated injections for patients [45]. Notably, ferritin, which stores iron in reticuloendothelial macrophages and hepatocytes, releases iron into the bloodstream via ferroportin when blood iron levels are inadequate. However, hepcidin, a peptide synthesized in the liver, binds to ferroportin, leading to the degradation of the hepcidin–ferroportin complex by lysosomes, hence controlling iron metabolism [46,47]. Additionally, hepcidin regulation has been documented to occur via the interleukin-6/signal transducers and activators of transcription 3 pathways, activated by inflammatory signaling mechanisms [48]. In CKD patients with anemia undergoing HD, inadequate iron availability and elevated hepcidin levels lead to ESA resistance in inflammatory conditions. In a study involving 30 persons undergoing HD transitioning from darbepoetin to roxadustat, a substantial reduction in hepcidin levels was observed from day 2 in the roxadustat cohort [49]. Meanwhile, HIF-PHIs were found to enhance iron availability by decreasing hepcidin levels in patients resistant to ESA [50]. Ferritin and transferrin indicate iron insufficiency and mobilization. TSAT and TIBC are both reliable indicators for diagnosing iron-deficient anemia. TIBC levels may be diminished in chronic inflammatory anemia, and a reduced TIBC signifies a worse prognosis in patients undergoing HD. TSAT indicates the availability of iron during erythropoiesis, and a reduction in TSAT is among the initial indicators of both absolute and functional iron shortage. Furthermore, research indicates that patients with low TSAT levels exhibit an elevated risk of cardiovascular disease as well as death relative to the ones with normal or high TSAT levels [51]. Our discovery was corroborated by prior experiments. An open-label phase III research study involving 2133 anemia patients with DD-CKD showed that roxadustat elevated SI and TIBC levels compared to epoetin alfa [52], indicating that roxadustat is superior to ESAs in managing iron metabolism among individuals suffering from CKD and undergoing HD.

Additionally, our meta-analysis evaluated the alteration of CRP levels from baseline. The results indicated that the CRP level was obviously lower in the group receiving roxadustat than in the ESA group, indirectly demonstrating the beneficial effect of roxadustat on inflammation. As previously stated, inflammation, a prevalent sign of anemia, was frequently observed to impact iron metabolism in patients with CKD undergoing HD, hence necessitating elevated dosages of ESAs and IV iron supplementation. Our prior meta-analysis demonstrated that, unlike ESAs, roxadustat sustained erythropoietic response irrespective of CRP levels in DD-CKD patients [53]. Certain correction studies indicate that roxadustat ameliorates anemia in patients undergoing HD regardless of baseline C-reactive protein levels, and its dosage requirements are less affected by inflammation compared to ESAs [54,55]. Recent reports indicated that roxadustat mitigates the inflammatory condition in patients undergoing HD. A self-controlled, single-center trial consisting of 30 patients exhibiting resistance to ESAs and undergoing maintenance HD demonstrated a reduction in inflammatory markers after a 3-month administration of roxadustat [56]. Meanwhile, researchers have suggested that roxadustat may inhibit the inflammatory response, including inflammatory indicators and the infiltration of inflammatory cells, by modulating metabolism, such as enhancing short-chain fatty acids or promoting angiogenesis [56,57]. Also, FG-4592 was documented to impede the activation of the inflammasome complex, hence safeguarding patients from acute kidney injury [58]. Further investigations are required to determine whether roxadustat decreases CRP levels and to elucidate the associated mechanisms.

AEs were classified using standardized terminology from the Adverse Event Dictionary of the Medical Dictionary for Regulatory Activities [59]. The occurrence of AEs was categorized by severity, MedDRA system organ categorization, preferred terminology, and the correlation of the AE with the study treatment. The definition of SAEs was derived from the National Cancer Institute’s Common Terminology Criteria for Adverse Events. Concerning safety, the findings demonstrated that there was no statistically important distinction in the occurrence of AEs and SAEs between the roxadustat cohort and the ESA cohort. However, in the post-marketing subgroup, the occurrence of AEs in the roxadustat cohort was markedly lower than that in the ESA cohort, aligning with the published meta-analysis of roxadustat in Chinese patients undergoing HD [18]. As an HIF-PHI, numerous trials have established the noninferiority of roxadustat in comparison to ESAs in patients with DD-CKD as well as in those with NDD-CKD, differing from vadadustat. While the latter was observed to be noninferior to DA when discussing cardiovascular safety profiles in DD-CKD patients, it did not meet the predefined criteria for noninferiority in patients with NDD-CKD [60,61]. In individuals with both NDD-CKD (ANDES, ALPS, and OLYMPUS) and DD-CKD (PYRENEES, SIERRAS, HIMALAYAS, and ROCKIES), the aggregated analysis of these phase III trials indicated that the occurrence of thromboembolic events was elevated in the roxadustat cohort compared to the ESA cohort. Thrombotic events, such as pulmonary embolism and deep vein thrombosis, could arise from elevated blood viscosity during anemia correction, necessitating caution in the clinical application of roxadustat, especially in individuals with a prior history of thromboembolic incidents. In the trials we covered, Akizawa et al. indicated that the occurrence of deep vein thrombosis was 1.3% (2/150) in the roxadustat cohort and 0% (0/152) in the DA cohort. Although the occurrence of SAEs in the roxadustat cohort was comparable to that in the ESA cohort among individuals suffering from CKD-related anemia and undergoing HD, the incidence of thromboembolism warrants further investigation.

Previous studies have shown the effectiveness and safety of roxadustat in managing anemia among patients with CKD [16,62]; however, the former study did not differentiate between hemodialysis and peritoneal dialysis, while the latter did not distinguish between dialysis and non-dialysis patients. Contrary to the published meta-analysis on individuals with CKD undergoing HD, our study encompassed a larger population, incorporating not only patients from China undergoing HD but also those from the US and Japan, thereby enhancing the applicability of the findings across diverse ethnic groups [18]. Furthermore, the clinical studies we included were not only post-marketing clinical trials but also phase II/III clinical trials, demonstrating that roxadustat is a potential alternative to ESAs in managing anemia among patients with CKD undergoing HD. Furthermore, the assessment of evidence quality was conducted in this study. These findings suggested that additional high-quality, large-sample RCTs are urgently needed in the future to furnish trustworthy data for the use of roxadustat in patients undergoing HD.

Before clinically generalizing the conclusions, certain limitations must be recognized. First, the methodological quality of RCTs included in the analysis was assessed as being low. None of the clinical studies specified whether allocation concealment was implemented. Only one RCT documented the implementation of a blinding strategy. All studies exhibited a high bias risk regarding the outcome assessment blinding due to the necessity of adjusting drug dosages based on Hb levels and indications linked to iron metabolism. Second, the heterogeneity test results for the majority of outcomes were significant in relation to populations and fundamental therapy. Despite the execution of subgroup and sensitivity analyses, the influence of confounding factors on heterogeneity remained unmitigated. Notably, the trial conduction phase was not the sole cause of heterogeneity. The Egger test did not reveal publication bias; however, the asymmetry observed in the funnel plot indicates significant heterogeneity among the studies analyzed. This finding necessitates a more cautious approach when interpreting the results. Third, the sample sizes of the RCTs were predominantly small, with most RCTs conducted in China, and the evidence grades for all outcomes were assessed as very poor, hence constraining the credibility and generalizability of the conclusions. Fourth, the observation duration was brief, necessitating ongoing monitoring and evaluation of roxadustat’s safety in long-term clinical applications. Only two of the RCTs included in this investigation documented SAEs, which were monitored for durations of 12 and 19 weeks, respectively. While the majority of research on DD-CKD has shown that roxadustat is noninferior to ESAs regarding safety profiles, certain data warrant concern. One study (PYRENEES) indicated a greater incidence of mortality in the roxadustat cohort (13.1, 95% CI 9.6 to 16.6) compared to the ESA cohort (6.8, 95% CI 4.3 to 9.2) with regard to the estimated Kaplan–Meier risk of death at 18 months [63], demonstrating the necessity for further assessment of roxadustat’s safety following prolonged clinical application. Future rigorous randomized double-blind placebo-controlled trials are required to evaluate the effectiveness as well as safety of roxadustat in managing anemia among individuals suffering from CKD and undergoing HD.

## 5. Conclusions

In this meta-analysis of post-marketing and phase II or III RCTs, roxadustat was superior to ESAs in improving SI, transferrin, and TIBC levels and reducing CRP levels. Roxadustat was not inferior to ESAs with regard to increasing Hb levels. And there were no significant variations in hepcidin, ferritin, and TSAT levels and the incidence of AEs and SAEs between roxadustat and ESAs. Aggregated analysis of phase II/III clinical trials demonstrated that roxadustat could increase serum Hb levels compared with ESAs in patients with CKD undergoing HD, without being affected by blood iron metabolism disorders and inflammation, which was confirmed by the post-marketing study. These findings suggest that roxadustat was well tolerated and a potent alternative to ESAs in patients with CKD undergoing HD.

## Figures and Tables

**Table 1:** The characteristic of patients.

Study ID	Country	Single/Multicenter	Phase	Sample Size (T/C)	Gender Ratio (Male/Female)	Age (Years) Mean ± SD	Baseline Hb (g/dL)	Interventions	Dosage of Medication	Control	Dosage of Medication	Study Duration
Akizawa 2020 [20]	Japan	multicenter	phase III	150/151	T:101/49 (2.06) C:107/44 (2.43)	T:64.6 ± 11.7 C:64.9 ± 10.1	T:11.02 ± 0.56 C:11.01 ± 0.60	roxadustat	70 mg or 100 mg, TIW	darbepoetin alfa	10–60 ug, QIW	24 weeks
Chen 2017 [25]	China	multicenter	phase II	74/22	T:45/29 (1.55) C:13/9 (1.44)	50.8 ± 12.6	10.7 ± 0.8	FG-4592	1.1–1.8 mg/kg, 1.5–2.3 mg/kg or 1.7–2.3 mg/kg, TIW	epoetin alfa	3000 to 20,000 IU/week	6 weeks
Provenzano 2016a [21]	USA	multicenter	phase II	41/13	T:27/14 (1.93) C:9/4 (2.25)	T:55.8 ± 13.4 C:59.5 ± 10.1	T:11.3 ± 0.6 C:11.5 ± 0.6	roxadustat	1.0 mg/kg, 1.5 mg/kg, 2.0 mg/kg, or 1.8 mg/kg, TIW	epoetin alfa	136.3 ± 47.7 IU/kg/wk	6 weeks
Provenzano 2016b [21]	USA	multicenter	phase II	67/23	T:45/22 (2.05) C:14/9 (1.56)	T:56.9 ± 12.1 C:57.0 ± 11.6	T:11.2 ± 0.7 C:11.2 ± 1.0	roxadustat	1.3 mg/kg; 2.0 mg/kg; 70–120–200 mg (1.68 ± 0.65 mg/kg, TIW)	epoetin alfa	173.4 ± 83.7 IU/kg/wk	19 weeks
Cai 2023 [22]	China	single-center	post-marketing	32/32	T:18/14 (1.29) C:17/15 (1.13)	T:51.98 ± 9.20 C:52.15 ± 9.25	T:132.5 ± 10.58 C:92.85 ± 9.15	roxadustat	70–120 mg, TIW	rhEPO	2000 IU, BIW/TIW	2 months
Cao 2022 [23]	China	single-center	post-marketing	27/28	T:14/13 (1.08) C:12/16 (0.75)	T:57.36 ± 7.75 C:58.94 ± 9.11	T:105.11 ± 10.24 C:98.55 ± 12.11	roxadustat	100 mg (45–60 kg) or 120 mg (≥60 kg), TIW	rhEPO	100–120 U/kg, TIW	3 months
Chen 2022 [24]	China	single-center	post-marketing	40/40	T:18/22 (0.82) C:20/20 (1.00)	T:53.40 ± 3.07 C:53.33 ± 3.11	T:103.55 ± 10.11 C:88.21 ± 5.68	roxadustat	100 mg (45–60 kg) or 120 mg (≥60 kg), TIW	rhEPO	100–120 IU/kg, BIW/TIW	3 months
Cheng 2021 [26]	China	single-center	post-marketing	33/32	T:20/13 (1.54) C:19/13 (1.46)	T:41.58 ± 2.15 C:41.52 ± 2.13	T:109.63 ± 7.05 C:95.37 ± 5.88	roxadustat	100 mg (<60 kg), or 120 mg (≥60 kg), TIW	rhEPO	Continued their previous doses	6 weeks
Hong 2023 [27]	China	single-center	post-marketing	50/50	T:28/22 (1.27) C:26/24 (1.08)	T:69.52 ± 8.97 C:70.12 ± 9.45	T:94.29 ± 12.41 C:85.54 ± 11.37	roxadustat	100 mg (≥45 kg and <60 kg), 120 mg (≥60 kg), TIW	rhEPO	100–150 IU/kg, TIW	12 weeks
Huang 2023 [28]	China	single-center	post-marketing	36/36	T:20/16 (1.25) C:24/12 (2.00)	T:51.36 ± 1.72 C:51.25 ± 1.31	T:112.91 ± 12.64 C:100.66 ± 9.44	roxadustat	100 mg (<60 kg), 120 mg (≥60 kg), TIW	rhEPO	80–150 U/kg, TIW	3 months
Jiang 2022 [29]	China	single-center	post-marketing	20/20	T:10/10 (1.00) C:11/8 (1.38)	T:50.1 ± 20.6 C:47.5 ± 17.1	T:95.38 ± 16.42 C:93.59 ± 14.73	roxadustat	100 mg (40–60 kg), 120 mg (>60 kg), TIW	rhEPO	10,000–20,000 U, QIW	12 weeks
Liang 2022 [30]	China	single-center	post-marketing	30/30	T:19/11 (1.73) C:18/12 (1.50)	T:49.98 ± 2.86 C:50.01 ± 2.19	T:102.83 ± 10.75 C:87.36 ± 8.12	roxadustat	100 mg (45–60 kg), 120 mg (≥60 kg), TIW	rhEPO	100–120 IU/kg, BIW/TIW	3 months
Liu 2022 [32]	China	single-center	post-marketing	25/25	T:13/12 (1.08) C:14/11 (1.27)	T:70.8 ± 4.15 C:71.64 ± 4.8	T:114.84 ± 10.04 C:109.04 ± 9.24	roxadustat	100 mg (45–60 kg) or 120 mg (≥60 kg), TIW	rhEPO	100–150 IU/kg, BIW/TIW	24 weeks
Liu 2023 [31]	China	single-center	post-marketing	60/60	T:33/27 (1.22) C:32/28 (1.14)	T:60.51 ± 7.66 C:60.36 ± 7.71	T:115.32 ± 10.32 C:109.31 ± 9.1	roxadustat	100 mg (45–60 kg) or 120 mg (≥60 kg), TIW	rhEPO	120 IU/kg, QIW	6 months
Sun 2022 [33]	China	single-center	post-marketing	36/34	T:14/22 (0.64) C:13/21 (0.62)	T:51.65 ± 3.53 C:51.29 ± 2.02	T:108.33 ± 14.11 C:100.86 ± 10.40	roxadustat	120 mg (≥60 kg), or 100 mg (<60 kg), TIW	rhEPO	Continued their previous doses	24 weeks
Tan 2021 [34]	China	single-center	post-marketing	34/36	T:18/16 (1.13) C:18/18 (1.00)	T:45.6 ± 7.1 C:47.6 ± 6.7	T:103.65 ± 10.01 C:87.12 ± 8.36	roxadustat	100 mg (40–60 kg) and following 120 mg, TIW	rhEPO	100 U/kg, TIW	3 months
Wang 2023 [35]	China	single-center	post-marketing	40/40	T:20/20 (1.00) C:21/19 (1.11)	T:52.85 ± 4.29 C:52.63 ± 4.17	T:104.76 ± 12.23 C:90.45 ± 12.70	roxadustat	100 mg (≤60 kg) or 120 mg (>60 kg), TIW	rhEPO	80–120 IU/kg, TIW	8 weeks
Wen 2022 [36]	China	single-center	post-marketing	62/62	T:34/28 (1.21) C:32/30 (1.07)	T:41.36 ± 8.92 C:43.43 ± 10.15	T:104.3 ± 10.42 C:87.58 ± 9.24	roxadustat	500 mg (40–60 kg) or 120 mg (>60 kg), BIW/TIW	rhEPO	100–150 U/kg, TIW	3 months
Yang 2022 [37]	China	single-center	post-marketing	30/30	T:21/9 (2.33) C:19/11 (1.73)	T:57.17 ± 8.62 C:56.42 ± 8.37	T:121.75 ± 10.08 C:108.38 ± 12.1	roxadustat	2 mg/kg, TIW	rhEPO	30–50 IU/kg, TIW	4 weeks
Zhang 2022 [38]	China	single-center	post-marketing	20/20	_	T:53.05 ± 14.85 C:58.10 ± 13.87	T:107.35 ± 20.72 C:100.75 ± 10.35	roxadustat	100 mg (<60 kg) or 120 mg (≥60 kg), TIW	rhEPO	100–150 IU/kg, TIW	12 weeks

TIW: three times weekly; BIW: twice weekly; QIW: once weekly; T: treatment; C: control; rhEPO: recombinant human erythropoietin.

**Table 2:** Results of meta-analysis.

Outcomes	Roxadustat	ESAs			
	n	n	I^2^	*p*	Meta-Analysis
Primary outcome					
∆Hb					
Post-marketing studies	575	575	93%	<0.00001	MD 1.35[0.92, 1.78]
Phase II/III studies	271	195	69%	0.38	MD 0.20[−0.25, 0.66]
Pooled results	846	770	96%	<0.00001	MD 1.17[0.71, 1.63]
Secondary outcomes					
∆SI					
Post-marketing studies	148	147	54%	0.003	SMD 0.53[0.18, 0.89]
Phase II/III studies	304	204	0%	<0.0001	SMD 0.39[0.20, 0.57]
Pooled results	452	351	23%	<0.00001	SMD 0.47[0.29, 0.65]
∆Hepcidin					
Post-marketing studies	96	98	0%	<0.0001	MD −26.87[−39.93, −13.81]
Phase II/III studies	289	200	53%	0.51	MD −11.20[−44.42, 22.01]
Pooled results	385	298	78%	0.11	MD −17.26[−38.66, 4.15]
∆Ferritin					
Post-marketing studies	442	443	98%	0.02	MD 38.25[6.87, 69.63]
Phase II/III studies	304	204	0%	0.12	MD 12.51[−3.26, 28.28]
Pooled results	746	647	98%	0.03	MD 30.99[3.01, 58.97]
∆Transferrin					
Post-marketing studies	306	305	87%	<0.00001	MD 0.60[0.42, 0.78]
Phase II/III studies	210	173	75%	<0.00001	MD 0.38[0.23, 0.52]
Pooled results	516	478	87%	<0.00001	MD 0.55[0.41, 0.69]
∆TIBC					
Post-marketing studies	85	85	89%	0.11	SMD 0.78[−0.17, 1.73]
Phase II/III studies	304	204	76%	<0.0001	SMD 0.98[0.49, 1.46]
Pooled results	389	289	80%	<0.0001	SMD 0.90[0.48, 1.31]
∆TSAT					
Post-marketing studies	483	483	97%	<0.00001	MD 7.15[4.39, 9.91]
Phase II/III studies	304	204	0%	0.06	MD 2.28[−0.06, 4.62]
Pooled results	787	687	96%	<0.00001	MD 6.22[3.78, 8.65]
∆CRP					
Post-marketing studies	124	125	26%	<0.0001	MD −2.10[−3.10, −1.10]
Safety					
AEs					
Post-marketing studies	483	483	10%	0.008	RR 0.60[0.41, 0.87]
Phase II/III studies	332	210	47%	0.46	RR 1.09[0.86, 1.38]
Pooled results	815	693	50%	0.17	RR 0.81[0.60, 1.09]
SAEs					
Phase II/III studies	258	188	0%	0.10	RR 1.43[0.94, 2.19]

**Table 3:** Results of Egger’ test.

Test	∆Hb	∆SI	∆Hepcidin	∆Ferritin	∆Transferrin	∆TIBC	∆TSAT	∆CRP
	*p* Value							
Egger’s test	0.131	0.051	0.153	0.852	0.06	0.174	0.942	0.87

**Table 4:** GRADE Evidence Profiles.

Outcomes	Certainty Assessment							№ of Patients		Effect		Certainty
	№ of Studies	Study Design	Risk of Bias	Inconsistency	Indirectness	Imprecision	Other Considerations	Continous	Placebo	Relative	Absolute	
										(95% CI)	(95% CI)	
∆Hb	19	randomised trials	very serious ^a^	serious ^b^	serious	not serious	none	846	770	-	MD 1.17 higher	⨁◯◯◯
											(0.71 higher to 1.63 higher)	Very low
∆SI	8	randomised trials	very serious ^a^	not serious	serious ^c^	not serious	none	452	351	-	SMD 0.47 higher	⨁◯◯◯
											(0.29 higher to 0.65 higher)	Very low
∆Hepcidin	6	randomised trials	very serious ^a^	serious ^b^	serious ^c^	serious ^e^	none	385	298	-	MD 17.26 lower	⨁◯◯◯
											(38.66 lower to 4.15 higher)	Very low
∆SF	17	randomised trials	very serious ^a^	very serious ^d^	serious ^c^	not serious	none	746	647	-	MD 30.99 higher	⨁◯◯◯
											(3.01 higher to 58.97 higher)	Very low
∆TRF	10	randomised trials	very serious ^a^	serious ^b^	serious ^c^	not serious	none	516	478	-	MD 0.55 higher	⨁◯◯◯
											(0.41 higher to 0.69 higher)	Very low
∆TIBC	7	randomised trials	very serious ^a^	serious ^b^	serious ^c^	not serious	none	389	289	-	SMD 0.9 higher	⨁◯◯◯
											(0.48 higher to 1.31 higher)	Very low
∆TSAT	17	randomised trials	very serious ^a^	very serious ^d^	serious ^c^	not serious	none	787	687	-	MD 6.22 higher	⨁◯◯◯
											(3.78 higher to 8.65 higher)	Very low
∆CRP	4	randomised trials	very serious ^a^	not serious	not serious	serious ^f^	none	124	125	-	MD 2.01 lower	⨁◯◯◯
											GRAD	Very low
AEs	17	randomised trials	very serious ^a^	not serious	serious ^c^	not serious	none	274/815 (33.6%)	232/693 (33.5%)	RR 0.81	64 fewer per 1000	⨁◯◯◯
										(0.60 to 1.09)	(from 134 fewer to 30 more)	Very low
SAEs	2	randomised trials	very serious ^a^	not serious	serious ^c^	serious ^g^	none	57/258 (22.1%)	28/188 (14.9%)	RR 1.43	64 more per 1000	⨁◯◯◯
										(0.94 to 2.19)	(from 9 fewer to 177 more)	Very low

CI: confidence interval; MD: mean difference; RR: risk ratio; SMD: standardized mean difference. ^a^ Only one article applied a blinding method, and none of the articles mentioned allocation concealment; ^b^ I^2^ > 50% and *p* < 0.1; ^c^ large racial disparities; ^d^ I^2^ > 90% and *p* < 0.1; ^e^ continuous-type ending data confidence intervals cross the null line; ^f^ sample size of continuous outcome data less than 400; ^g^ confidence intervals for dichotomous ending data crossing the null line while crossing 1.25.

## Data Availability

All relevant data are within the manuscript and Supplementary Materials.

## Supplementary Materials

The following supporting information can be downloaded at https://www.mdpi.com/article/10.3390/toxics12120846/s1, Figure S1: The forest graph of Hb (g/dL); Figure S2: The forest graph of ∆Hb after removing 1 RCT; Figure S3: The forest graph of ∆SI (umol/L); Figure S4: The forest graph of ∆SI after removing 1 RCT; Figure S5: The forest graph of ∆hepcidin after removing 1 RCT; Figure S6: The forest graph of ∆TIBC (umol/L). The retrieval strategy.

## References

[1] Matsushita K., Ballew S.H., Wang A.Y., Kalyesubula R., Schaeffner E., Agarwal R. (2022). Epidemiology and risk of cardiovascular disease in populations with chronic kidney disease. Nat. Rev. Nephrol..

[2] Babitt J.L., Lin H.Y. (2012). Mechanisms of anemia in CKD. J. Am. Soc. Nephrol..

[3] Stauffer M.E., Fan T. (2014). Prevalence of anemia in chronic kidney disease in the United States. PLoS ONE.

[4] Locatelli F., Covic A., Eckardt K.U., Wiecek A., Vanholder R. (2009). Anaemia management in patients with chronic kidney disease: A position statement by the Anaemia Working Group of European Renal Best Practice (ERBP). Nephrol. Dial. Transpl..

[5] Babitt J.L., Eisenga M.F., Haase V.H., Kshirsagar A.V., Levin A., Locatelli F., Małyszko J., Swinkels D.W., Tarng D.C., Cheung M. (2021). Controversies in optimal anemia management: Conclusions from a Kidney Disease: Improving Global Outcomes (KDIGO) Conference. Kidney Int..

[6] Johansen K.L., Finkelstein F.O., Revicki D.A., Evans C., Wan S., Gitlin M., Agodoa I.L. (2012). Systematic review of the impact of erythropoiesis-stimulating agents on fatigue in dialysis patients. Nephrol. Dial. Transpl..

[7] Sakaguchi Y., Hamano T., Wada A., Masakane I. (2019). Types of Erythropoietin-Stimulating Agents and Mortality among Patients Undergoing Hemodialysis. J. Am. Soc. Nephrol..

[8] Minutolo R., Garofalo C., Chiodini P., Aucella F., Del Vecchio L., Locatelli F., Scaglione F., De Nicola L. (2021). Types of erythropoiesis-stimulating agents and risk of end-stage kidney disease and death in patients with non-dialysis chronic kidney disease. Nephrol. Dial. Transpl..

[9] Singh A.K., Szczech L., Tang K.L., Barnhart H., Sapp S., Wolfson M., Reddan D. (2006). Correction of anemia with epoetin alfa in chronic kidney disease. N. Engl. J. Med..

[10] Weir M.R. (2021). Managing Anemia across the Stages of Kidney Disease in Those Hyporesponsive to Erythropoiesis-Stimulating Agents. Am. J. Nephrol..

[11] Portolés J., Martín L., Broseta J.J., Cases A. (2021). Anemia in Chronic Kidney Disease: From Pathophysiology and Current Treatments, to Future Agents. Front. Med..

[12] Umanath K., Jalal D.I., Greco B.A., Umeukeje E.M., Reisin E., Manley J., Zeig S., Negoi D.G., Hiremath A.N., Blumenthal S.S. (2015). Ferric Citrate Reduces Intravenous Iron and Erythropoiesis-Stimulating Agent Use in ESRD. J. Am. Soc. Nephrol..

[13] Fishbane S., Block G.A., Loram L., Neylan J., Pergola P.E., Uhlig K., Chertow G.M. (2017). Effects of Ferric Citrate in Patients with Nondialysis-Dependent CKD and Iron Deficiency Anemia. J. Am. Soc. Nephrol..

[14] Gupta N., Wish J.B. (2017). Hypoxia-Inducible Factor Prolyl Hydroxylase Inhibitors: A Potential New Treatment for Anemia in Patients With CKD. Am. J. Kidney Dis..

[15] Dhillon S. (2019). Roxadustat: First Global Approval. Drugs.

[16] Zheng Q., Yang H., Fu X., Huang Y., Wei R., Wang Y., Liu Y.N., Liu W.J. (2021). The efficacy and safety of roxadustat for anemia in patients with chronic kidney disease: A meta-analysis. Nephrol. Dial. Transpl..

[17] Chen N., Hao C., Liu B.C., Lin H., Wang C., Xing C., Liang X., Jiang G., Liu Z., Li X. (2019). Roxadustat Treatment for Anemia in Patients Undergoing Long-Term Dialysis. N. Engl. J. Med..

[18] Liang Q., Li X., Niu Q., Zhao H., Zuo L. (2023). Efficacy and Safety of Roxadustat in Chinese Hemodialysis Patients: A Systematic Review and Meta-Analysis. J. Clin. Med..

[19] Page M.J., McKenzie J.E., Bossuyt P.M., Boutron I., Hoffmann T.C., Mulrow C.D., Shamseer L., Tetzlaff J.M., Akl E.A., Brennan S.E. (2021). The PRISMA 2020 statement: An updated guideline for reporting systematic reviews. BMJ.

[20] Akizawa T., Iwasaki M., Yamaguchi Y., Majikawa Y., Reusch M. (2020). Phase 3, Randomized, Double-Blind, Active-Comparator (Darbepoetin Alfa) Study of Oral Roxadustat in CKD Patients with Anemia on Hemodialysis in Japan. J. Am. Soc. Nephrol..

[21] Provenzano R., Besarab A., Wright S., Dua S., Zeig S., Nguyen P., Poole L., Saikali K.G., Saha G., Hemmerich S. (2016). Roxadustat (FG-4592) Versus Epoetin Alfa for Anemia in Patients Receiving Maintenance Hemodialysis: A Phase 2, Randomized, 6- to 19-Week, Open-Label, Active-Comparator, Dose-Ranging, Safety and Exploratory Efficacy Study. Am. J. Kidney Dis..

[22] Cai Z., Fu T. (2023). Study on the efficacy and mechanism of action of roxadustat in patients with uremia on maintenance hemodialysis with renal anemia. Chin. Foreign Med. Res..

[23] Cao B., Wang F., Mo Y. (2022). Comparison of the clinical effects of roxadustat and erythropoietin in the treatment of renal anemia in patients on maintenance hemodialysis. Chin. J. Clin. Ration. Drug Use.

[24] Chen N. (2022). Analysis of the clinical therapeutic effect of roxadustat on renal anemia in hemodialysis patients. China Foreign Med. Treat..

[25] Chen N., Qian J., Chen J., Yu X., Mei C., Hao C., Jiang G., Lin H., Zhang X., Zuo L. (2017). Phase 2 studies of oral hypoxia-inducible factor prolyl hydroxylase inhibitor FG-4592 for treatment of anemia in China. Nephrol. Dial. Transplant..

[26] Cheng H., Wei Z., Shi H., Li G., Wu X. (2021). Efficacy of roxadustat in the treatment of refractory renal anemia in patients undergoing maintenance hemodialysis. J. Clin. Med. Pract..

[27] Hong Y., Wang Z., Peng H., Xu F. (2023). Efficacy of roxadustat versus erythropoietin in the treatment of renal anemia combined with hemodialysis in the elderly and the effect on cardiovascular indicators. Chin. J. Gerontol..

[28] Huang H., Wei F. (2023). Comparison of oral roxadustat and recombinant human erythropoietin in the treatment of patients with renal anemia combined with chronic maintenance dialysis. China Mod. Med..

[29] Jiang F., Hong D., Du Y., Gan C., Chen Q., Guan X., Deng F. (2022). Observation on the efficacy and safety of roxadustat in the treatment of hemodialysis patients with renal anemia. Pract. Pharm. Clin. Remedies.

[30] Liang Y., Li W., Wu M. (2022). Effect of roxadustat on anemia-related indicators and iron metabolism indicators in patients with renal anemia combined with hemodialysis. Int. J. Transplant. Hemopurif..

[31] Liu Y., He Y., Feng L., Wu L., Lai Y., Hong R. (2023). Effectiveness of roxadustat capsules in the treatment of renal anemia in patients undergoing maintenance hemodialysis and the effect on biochemical indexes. MedicaI Innov. China.

[32] Liu Y., Liu L., Li Z., Wan Y., Bai Y. (2022). Efficacy and safety of roxadustat in the treatment of renal anemia in elderly patients on maintenance hemodialysis. Anhui Med. J..

[33] Sun W., Tong Y., Tang Y., Wu J., He H., Xu X. (2022). Comparison of the efficacy of roxadustat and recombinant human erythropoietin in the treatment of renal anemia in patients on maintenance hemodialysis. Int. J. Urol. Nephrol..

[34] Tan P., Luo L., Deng W., Tan X. (2021). A comparative study of the clinical efficacy of roxadustat and recombinant human erythropoietin in the treatment of renal anemia on hemodialysis. Chin. J. Clin. Ration. Drug Use.

[35] Wang L., Yang M., Hou J., Wang L. (2023). Evaluation of the efficacy and safety of roxadustat in hemodialysis patients with renal anemia based on the maximum difference between pre- and post-treatment changes in erythropoietin and interleukin-6 levels and hemoglobin. J. Clin. Nephrol..

[36] Wen C., Zhang H., Qin X., Yang J. (2022). Comparison of the clinical effects of roxadustat and erythropoietin in the treatment of patients with renal anemia complicated by maintenance hemodialysis. Intern. Med..

[37] Yang L. (2022). Analysis of the efficacy of roxadustat in the treatment of renal anemia in patients undergoing maintenance hemodialysis. Mod. Diagn. Treat..

[38] Zhang Y., Huo j., Zhou h., Qiu x., Tu Y. (2022). Efficacy and safety of roxadustat capsules versus recombinant human erythropoietin injection in the treatment of renal anemia on maintenance hemodialysis. Int. J. Urol. Nephrol..

[39] Del Balzo U., Signore P.E., Walkinshaw G., Seeley T.W., Brenner M.C., Wang Q., Guo G., Arend M.P., Flippin L.A., Chow F.A. (2020). Nonclinical Characterization of the Hypoxia-Inducible Factor Prolyl Hydroxylase Inhibitor Roxadustat, a Novel Treatment of Anemia of Chronic Kidney Disease. J. Pharmacol. Exp. Ther..

[40] Groenendaal-van de Meent D., Kerbusch V., Kaspera R., Barroso-Fernandez B., Galletti P., Klein G.K., den Adel M. (2021). Effect of Kidney Function and Dialysis on the Pharmacokinetics and Pharmacodynamics of Roxadustat, an Oral Hypoxia-Inducible Factor Prolyl Hydroxylase Inhibitor. Eur. J. Drug Metab. Pharmacokinet..

[41] Provenzano R., Tumlin J., Zabaneh R., Chou J., Hemmerich S., Neff T.B., Yu K.P. (2020). Oral Hypoxia-Inducible Factor Prolyl Hydroxylase Inhibitor Roxadustat (FG-4592) for Treatment of Anemia in Chronic Kidney Disease: A Placebo-Controlled Study of Pharmacokinetic and Pharmacodynamic Profiles in Hemodialysis Patients. J. Clin. Pharmacol..

[42] Zhang L., Hou J., Li J., Su S.S., Xue S. (2021). Roxadustat for the treatment of anemia in patients with chronic kidney diseases: A meta-analysis. Aging.

[43] Liu C., Fu Z., Jiang J., Chi K., Geng X., Mao Z., Song C., Sun G., Hong Q., Cai G. (2021). Safety and Efficacy of Roxadustat for Anemia in Patients With Chronic Kidney Disease: A Meta-Analysis and Trial Sequential Analysis. Front. Med..

[44] Gafter-Gvili A., Schechter A., Rozen-Zvi B. (2019). Iron Deficiency Anemia in Chronic Kidney Disease. Acta. Haematol..

[45] Schaefer B., Meindl E., Wagner S., Tilg H., Zoller H. (2020). Intravenous iron supplementation therapy. Mol. Aspects Med..

[46] Drakesmith H., Nemeth E., Ganz T. (2015). Ironing out Ferroportin. Cell Metab..

[47] Ganz T. (2019). Erythropoietic regulators of iron metabolism. Free Radic. Biol. Med..

[48] Wrighting D.M., Andrews N.C. (2006). Interleukin-6 induces hepcidin expression through STAT3. Blood.

[49] Ogawa C., Tsuchiya K., Tomosugi N., Maeda K. (2020). A Hypoxia-Inducible Factor Stabilizer Improves Hematopoiesis and Iron Metabolism Early after Administration to Treat Anemia in Hemodialysis Patients. Int. J. Mol. Sci..

[50] Ogawa C., Tsuchiya K., Maeda K. (2023). Hypoxia-Inducible Factor Prolyl Hydroxylase Inhibitors and Iron Metabolism. Int. J. Mol. Sci..

[51] Fu Z., Geng X., Chi K., Song C., Wu D., Liu C., Hong Q. (2022). Efficacy and Safety of Daprodustat Vs rhEPO for Anemia in Patients With Chronic Kidney Disease: A Meta-Analysis and Trial Sequential Analysis. Front. Pharmacol..

[52] Fishbane S., Pollock C.A., El-Shahawy M., Escudero E.T., Rastogi A., Van B.P., Frison L., Houser M., Pola M., Little D.J. (2022). Roxadustat Versus Epoetin Alfa for Treating Anemia in Patients with Chronic Kidney Disease on Dialysis: Results from the Randomized Phase 3 ROCKIES Study. J. Am. Soc. Nephrol..

[53] Zheng Q., Zhang P., Yang H., Geng Y., Tang J., Kang Y., Qi A., Li S. (2023). Effects of hypoxia-inducible factor prolyl hydroxylase inhibitors versus erythropoiesis-stimulating agents on iron metabolism and inflammation in patients undergoing dialysis: A systematic review and meta-analysis. Heliyon.

[54] Locatelli F., Del Vecchio L. (2022). Hypoxia-Inducible Factor-Prolyl Hydroxyl Domain Inhibitors: From Theoretical Superiority to Clinical Noninferiority Compared with Current ESAs?. J. Am. Soc. Nephrol..

[55] Besarab A., Chernyavskaya E., Motylev I., Shutov E., Kumbar L.M., Gurevich K., Chan D.T., Leong R., Poole L., Zhong M. (2016). Roxadustat (FG-4592): Correction of Anemia in Incident Dialysis Patients. J. Am. Soc. Nephrol..

[56] Zhao X.N., Liu S.X., Wang Z.Z., Zhang S., You L.L. (2023). Roxadustat alleviates the inflammatory status in patients receiving maintenance hemodialysis with erythropoiesis-stimulating agent resistance by increasing the short-chain fatty acids producing gut bacteria. Eur. J. Med. Res..

[57] Miao A.F., Liang J.X., Yao L., Han J.L., Zhou L.J. (2021). Hypoxia-inducible factor prolyl hydroxylase inhibitor roxadustat (FG-4592) protects against renal ischemia/reperfusion injury by inhibiting inflammation. Ren. Fail..

[58] Yang H., Wu Y., Cheng M., Zhang M., Qiu X., Liu S., Zhang M. (2023). Roxadustat (FG-4592) protects against ischaemia-induced acute kidney injury via improving CD73 and decreasing AIM2 inflammasome activation. Nephrol. Dial. Transpl..

[59] Besarab A., Provenzano R., Hertel J., Zabaneh R., Klaus S.J., Lee T., Leong R., Hemmerich S., Yu K.H., Neff T.B. (2015). Randomized placebo-controlled dose-ranging and pharmacodynamics study of roxadustat (FG-4592) to treat anemia in nondialysis-dependent chronic kidney disease (NDD-CKD) patients. Nephrol. Dial. Transpl..

[60] Eckardt K.U., Agarwal R., Aswad A., Awad A., Block G.A., Bacci M.R., Farag Y.M.K., Fishbane S., Hubert H., Jardine A. (2021). Safety and Efficacy of Vadadustat for Anemia in Patients Undergoing Dialysis. N. Engl. J. Med..

[61] Chertow G.M., Pergola P.E., Farag Y.M.K., Agarwal R., Arnold S., Bako G., Block G.A., Burke S., Castillo F.P., Jardine A.G. (2021). Vadadustat in Patients with Anemia and Non-Dialysis-Dependent CKD. N. Engl. J. Med..

[62] Wang L., Yin H., Yang L., Zhang F., Wang S., Liao D. (2022). The Efficacy and Safety of Roxadustat for Anemia in Patients with Chronic Kidney Disease: A Meta-Analysis. Front. Pharmacol..

[63] Csiky B., Schömig M., Esposito C., Barratt J., Reusch M., Valluri U., Sulowicz W. (2021). Roxadustat for the Maintenance Treatment of Anemia in Patients with End-Stage Kidney Disease on Stable Dialysis: A European Phase 3, Randomized, Open-Label, Active-Controlled Study (PYRENEES). Adv. Ther..

