# Supplementary material for: The Efficacy and Safety of Roxadustat for Anemia in Hemodialysis Patients with Chronic Kidney Disease: A Meta-Analysis of Randomized Controlled Trials
Source: Toxics. 2024 Nov 25;12(12):846. doi: 10.3390/toxics12120846 (PMC11679264; doi:10.3390/toxics12120846)
Supplement: Supplementary file 1 [file toxics-12-00846-s001.zip › toxics-3280304-supplementary.pdf]

Supplementary materials

Meta-analysis:

S1. Hb

Study or Subgroup	Experimental Mean	Experimental SD	Experimental Total	Control Mean	Control SD	Control Total	Weight	Mean Difference IV, Random, 95% CI
**3.9.1 Post-marketing**								
Cai 2023	13.25	1.058	32	9.285	0.915	32	5.1%	3.96 [3.48, 4.45]
Cao 2022	10.511	1.024	27	9.855	1.211	28	4.9%	0.66 [0.06, 1.25]
Chen 2022	10.355	1.011	40	8.821	0.568	40	5.2%	1.53 [1.17, 1.89]
Cheng 2021	10.963	0.705	33	9.537	0.588	32	5.3%	1.43 [1.11, 1.74]
Hong 2023	9.429	1.241	50	8.554	1.137	50	5.1%	0.88 [0.41, 1.34]
Huang 2023	11.291	1.264	36	10.066	0.944	36	5.0%	1.22 [0.71, 1.74]
Jiang 2022	9.538	1.642	20	9.359	1.473	20	4.1%	0.18 [-0.79, 1.15]
Liang 2022	10.283	1.075	30	8.736	0.812	30	5.1%	1.55 [1.06, 2.03]
Liu 2022	11.484	1.004	25	10.904	0.924	25	5.0%	0.58 [0.05, 1.11]
Liu 2023	11.532	1.032	60	10.931	0.91	60	5.3%	0.60 [0.25, 0.95]
Sun 2022	10.833	1.411	36	10.086	1.04	34	4.9%	0.75 [0.17, 1.33]
Tan 2021	10.365	1.001	34	8.712	0.836	36	5.1%	1.65 [1.22, 2.09]
Wang 2023	10.476	1.223	40	9.045	1.27	40	5.0%	1.43 [0.88, 1.98]
Wen 2022	10.43	1.042	62	8.758	0.924	62	5.3%	1.67 [1.33, 2.02]
Yang 2022	12.175	1.008	30	10.838	1.21	30	4.9%	1.34 [0.77, 1.90]
Zhang 2022	10.43	0.52	54	9.066	0.512	53	5.4%	1.36 [1.17, 1.56]
**Subtotal (95% CI)**			**609**			**608**	**80.6%**	**1.32 [0.98, 1.67]**
Heterogeneity: Tau² = 0.44; Chi² = 163.81, df = 15 (P < 0.00001); I² = 91%								
Test for overall effect: Z = 7.47 (P < 0.00001)								
**3.9.2 Phase II/III**								
Akizawa 2020	11	0.6	150	10.95	0.63	151	5.4%	0.05 [-0.09, 0.19]
Chen 2017	11.54	1.98	60	10.77	1.96	22	4.1%	0.77 [-0.19, 1.73]
Provenzano 2016a	11.6	0.7	33	10.5	0.8	9	4.9%	1.10 [0.53, 1.67]
Provenzano 2016b	10.7	0.9	61	10.7	1.3	22	4.9%	0.00 [-0.59, 0.59]
**Subtotal (95% CI)**			**304**			**204**	**19.4%**	**0.42 [-0.13, 0.96]**
Heterogeneity: Tau² = 0.22; Chi² = 14.06, df = 3 (P = 0.003); I² = 79%								
Test for overall effect: Z = 1.51 (P = 0.13)								
**Total (95% CI)**			**913**			**812**	**100.0%**	**1.15 [0.75, 1.55]**
Heterogeneity: Tau² = 0.75; Chi² = 403.41, df = 19 (P < 0.00001); I² = 95%								
Test for overall effect: Z = 5.69 (P < 0.00001)								
Test for subgroup differences: Chi² = 7.52, df = 1 (P = 0.006), I² = 86.7%								

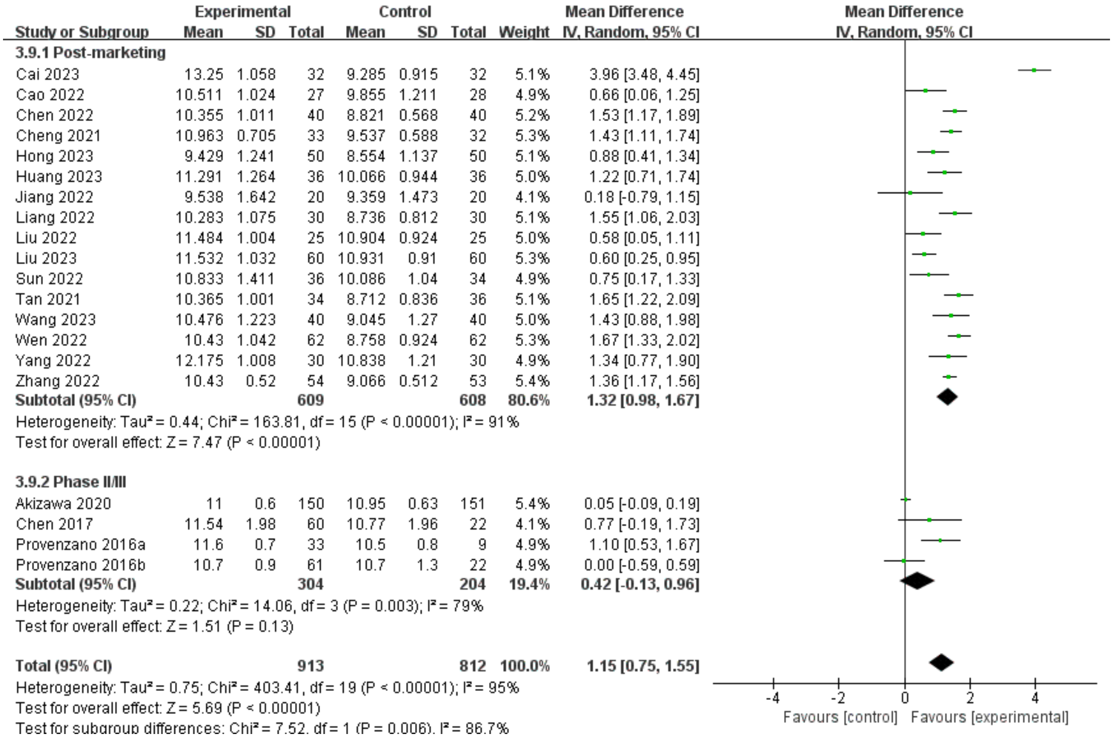

Figure S1 The forest graph of Hb (g/dL).

S2. ΔHb sensitivity analysis

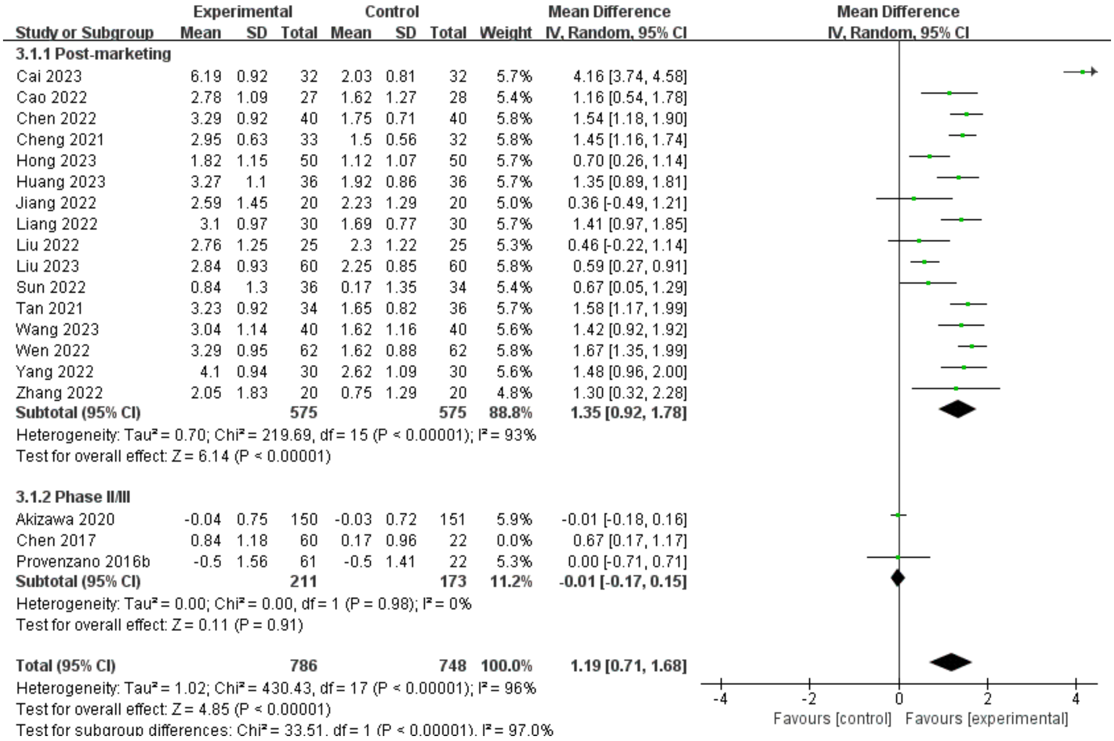

Figure S2 The forest graph of ΔHb after removing 1 RCT.

S3. ΔSI

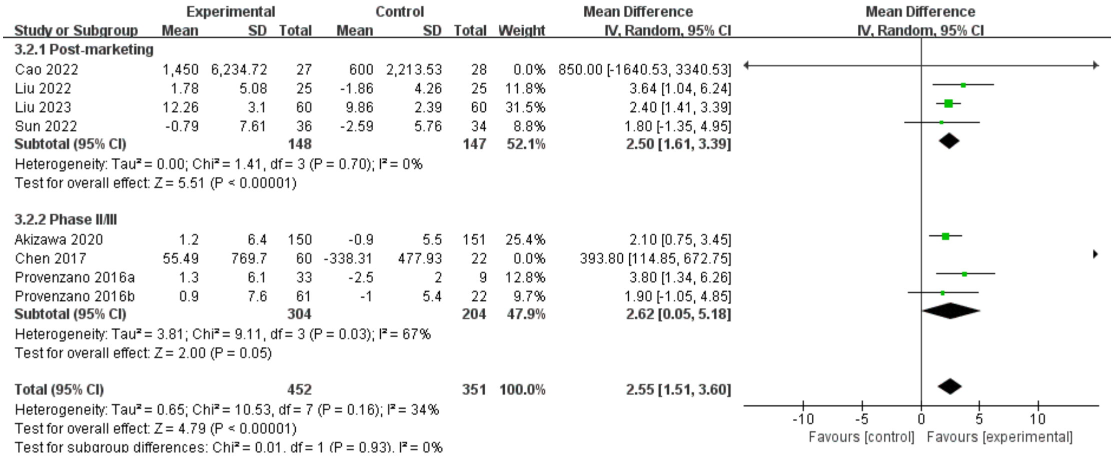

Figure S3 The forest graph of ΔSI (umol/L).

S4. ΔSI sensitivity analysis

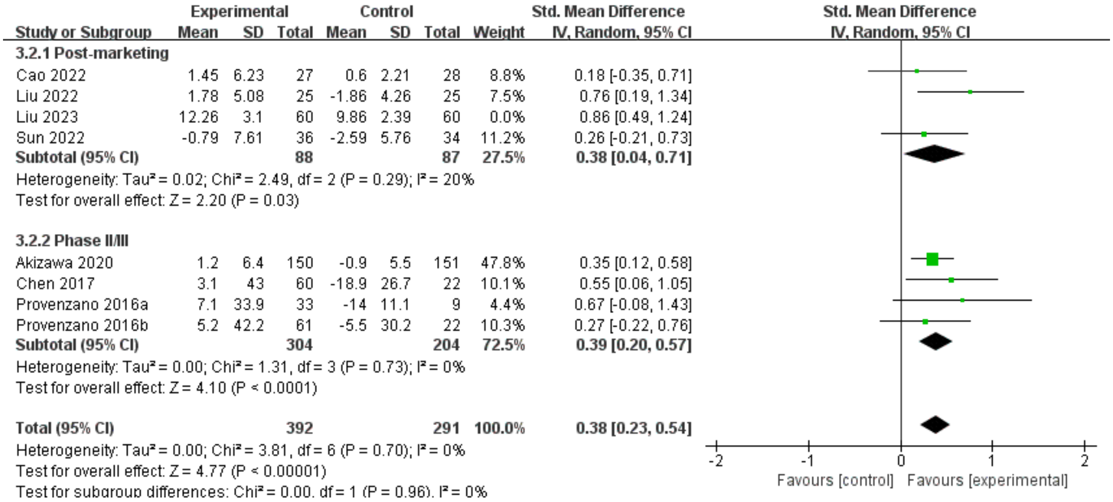

Figure S4 The forest graph of ΔSI after removing 1 RCT.

S5. Δhepcidin sensitivity analysis

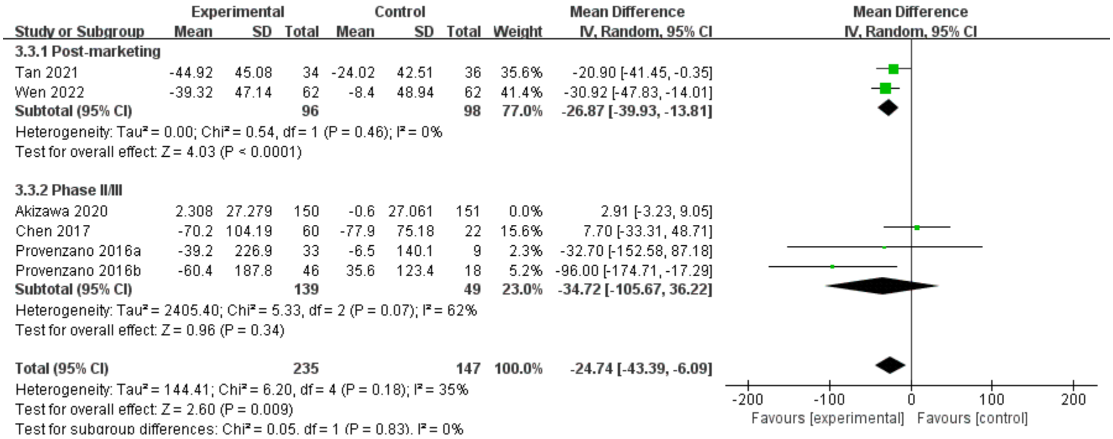

Figure S5 The forest graph of Δhepcidin after removing 1 RCT.

S6. ΔTIBC

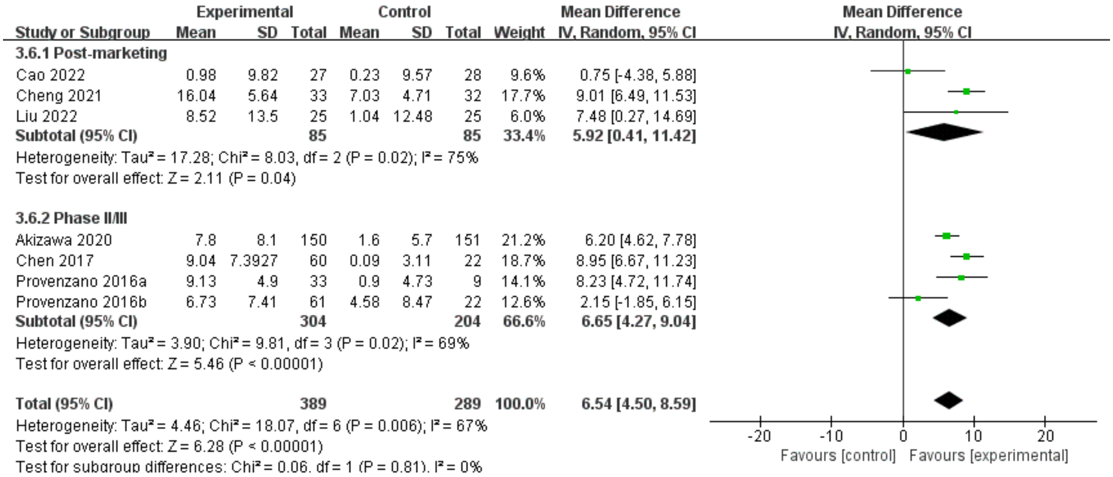

Figure S6 The forest graph of ΔTIBC (umol/L).

.The retrieval strategy

PubMed:

#1 ("Renal Insufficiency, Chronic"[Mesh]) OR (Chronic Renal Insufficiencies[Title/Abstract] OR Renal Insufficiencies, Chronic[Title/Abstract] OR Chronic Renal Insufficiency[Title/Abstract] OR Kidney Insufficiency, Chronic[Title/Abstract] OR Chronic Kidney Insufficiency[Title/Abstract] OR Chronic Kidney Insufficiencies[Title/Abstract] OR Kidney Insufficiencies, Chronic[Title/Abstract] OR Chronic Kidney Diseases[Title/Abstract] OR Chronic Kidney Disease[Title/Abstract] OR Disease, Chronic Kidney[Title/Abstract] OR Diseases, Chronic Kidney[Title/Abstract] OR Kidney Disease, Chronic[Title/Abstract] OR Kidney Diseases, Chronic[Title/Abstract] OR Chronic Renal Diseases[Title/Abstract] OR Chronic Renal Disease[Title/Abstract] OR Disease, Chronic Renal[Title/Abstract] OR Diseases, Chronic Renal[Title/Abstract] OR Renal Disease, Chronic[Title/Abstract] OR Renal Diseases, Chronic[Title/Abstract])

#2 ("Kidney Failure, Chronic"[Mesh]) OR (End-Stage Kidney Disease[Title/Abstract] OR Disease, End-Stage Kidney[Title/Abstract] OR End Stage Kidney Disease[Title/Abstract] OR Kidney Disease, End-Stage[Title/Abstract] OR Chronic Kidney Failure[Title/Abstract] OR End-Stage Renal Disease[Title/Abstract] OR Disease, End-Stage Renal[Title/Abstract] OR End Stage Renal Disease[Title/Abstract] OR Renal Disease, End-Stage[Title/Abstract] OR Renal Disease, End Stage[Title/Abstract] OR Renal Failure, End-Stage[Title/Abstract] OR End-Stage Renal Failure[Title/Abstract] OR Renal Failure, End Stage[Title/Abstract] OR Renal Failure, Chronic[Title/Abstract] OR Chronic Renal Failure[Title/Abstract] OR ESRD[Title/Abstract])

#3 ("Anemia"[Mesh]) OR (Anemias[Title/Abstract])

#4 ("Renal Dialysis"[Mesh]) OR (Dialyses, Renal[Title/Abstract] OR Renal Dialyses[Title/Abstract] OR Dialysis, Renal[Title/Abstract] OR Hemodialysis[Title/Abstract] OR Hemodialyses[Title/Abstract] OR Dialysis, Extracorporeal[Title/Abstract] OR Dialyses, Extracorporeal[Title/Abstract] OR

Extracorporeal Dialyses[Title/Abstract] OR Extracorporeal Dialysis[Title/Abstract])
#5 Hypoxia-inducible factor prolyl hydroxylase inhibitors[Title/Abstract] OR HIF-PHIs[Title/Abstract] OR roxadustat[Title/Abstract] OR FG-4592[Title/Abstract]
#6 (randomized controlled trial[pt] OR controlled clinical trial[pt] OR randomized[tiab] OR placebo[tiab] OR clinical trials as topic[mesh:noexp] OR randomly[tiab] OR trial[ti]) NOT (animals[mh] NOT (humans[mh] AND animals[mh]))
#7 #1 OR #2
#8 #3 AND #4 AND #5 AND #6 AND #7
Web of Science:
#1 TS=(Renal Insufficiency, Chronic OR Chronic Renal Insufficiencies OR Renal Insufficiencies, Chronic OR Chronic Renal Insufficiency OR Kidney Insufficiency, Chronic OR Chronic Kidney Insufficiency OR Chronic Kidney Insufficiencies OR Kidney Insufficiencies, Chronic OR Chronic Kidney Diseases OR Chronic Kidney Disease OR Disease, Chronic Kidney OR Diseases, Chronic Kidney OR Kidney Disease, Chronic OR Kidney Diseases, Chronic OR Chronic Renal Diseases OR Chronic Renal Disease OR Disease, Chronic Renal OR Diseases, Chronic Renal OR Renal Disease, Chronic OR Renal Diseases, Chronic OR Kidney Failure, Chronic OR End-Stage Kidney Disease OR Disease, End-Stage Kidney OR End Stage Kidney Disease OR Kidney Disease, End-Stage OR Chronic Kidney Failure OR End-Stage Renal Disease OR Disease, End-Stage Renal OR End Stage Renal Disease OR Renal Disease, End-Stage OR Renal Disease, End Stage OR Renal Failure, End-Stage OR End-Stage Renal Failure OR Renal Failure, End Stage OR Renal Failure, Chronic OR Chronic Renal Failure OR ESRD)
#2 TS=(Renal Dialysis OR Dialyses, Renal OR Renal Dialyses OR Dialysis, Renal OR Hemodialysis OR Hemodialyses OR Dialysis, Extracorporeal OR Dialyses, Extracorporeal OR Extracorporeal Dialyses OR Extracorporeal Dialysis)
#3 TS=(Anemia OR Anemias)
#4 TS=(Hypoxia-inducible factor prolyl hydroxylase inhibitors OR HIF-PHIs OR roxadustat OR FG-4592)
#5 TS=((randomized controlled trial OR controlled clinical trial OR randomized OR placebo OR randomly OR trial) NOT (animals NOT (humans AND animals)))
#6 (#1 AND #2 AND #3 AND #4 AND #5) AND Article or Early Access or Proceeding Paper (Document Types)
Cochrane Library:
#1 MeSH descriptor: [Renal Insufficiency, Chronic] explode all trees
#2 MeSH descriptor: [Kidney Failure, Chronic] explode all trees
#3 MeSH descriptor: [Anemia] explode all trees
#4 MeSH descriptor: [Dialysis] explode all trees
#5 (renal insufficiency, chronic):ti,ab,kw OR (kidney failure, chronic):ti,ab,kw AND (anemia):ti,ab,kw AND (dialysis):ti,ab,kw
#6 #1 OR #2 AND #3 AND #4 OR #5
#7 (Hypoxia-inducible factor prolyl hydroxylase inhibitors):ti,ab,kw OR (HIF-PHIs):ti,ab,kw OR (roxadustat):ti,ab,kw OR (FG-4592):ti,ab,kw

#8 #6 AND #7 in trials

Embase:

#1 'chronic kidney failure'/exp OR 'end stage renal disease'/exp

#2 'renal insufficiency, chronic':ti,ab,kw OR 'chronic renal insufficiencies':ti,ab,kw OR 'renal insufficiencies, chronic':ti,ab,kw OR 'chronic renal insufficiency':ti,ab,kw OR 'kidney insufficiency, chronic':ti,ab,kw OR 'chronic kidney insufficiency':ti,ab,kw OR 'chronic kidney insufficiencies':ti,ab,kw OR 'kidney insufficiencies, chronic':ti,ab,kw OR 'chronic kidney diseases':ti,ab,kw OR 'chronic kidney disease':ti,ab,kw OR 'disease, chronic kidney':ti,ab,kw OR 'diseases, chronic kidney':ti,ab,kw OR 'kidney disease, chronic':ti,ab,kw OR 'kidney diseases, chronic':ti,ab,kw OR 'chronic renal diseases':ti,ab,kw OR 'chronic renal disease':ti,ab,kw OR 'disease, chronic renal':ti,ab,kw OR 'diseases, chronic renal':ti,ab,kw OR 'renal disease, chronic':ti,ab,kw OR 'renal diseases, chronic':ti,ab,kw

#3 'kidney failure, chronic':ti,ab,kw OR 'end-stage kidney disease':ti,ab,kw OR 'disease, end-stage kidney':ti,ab,kw OR 'end stage kidney disease':ti,ab,kw OR 'kidney disease, end-stage':ti,ab,kw OR 'chronic kidney failure':ti,ab,kw OR 'end-stage renal disease':ti,ab,kw OR 'disease, end-stage renal':ti,ab,kw OR 'end stage renal disease':ti,ab,kw OR 'renal disease, end-stage':ti,ab,kw OR 'renal disease, end stage':ti,ab,kw OR 'renal failure, end-stage':ti,ab,kw OR 'end-stage renal failure':ti,ab,kw OR 'renal failure, end stage':ti,ab,kw OR 'renal failure, chronic':ti,ab,kw OR 'chronic renal failure':ti,ab,kw OR esrd:ti,ab,kw

#4 'anemia'/exp

#5 anemia:ti,ab,kw OR anemias:ti,ab,kw

#6 'dialysis'/exp

#7 'renal dialysis':ti,ab,kw OR 'dialyses, renal':ti,ab,kw OR 'renal dialyses':ti,ab,kw OR 'dialysis, renal':ti,ab,kw OR hemodialysis:ti,ab,kw OR hemodialyses:ti,ab,kw OR 'dialysis, extracorporeal':ti,ab,kw OR 'dialyses, extracorporeal':ti,ab,kw OR 'extracorporeal dialyses':ti,ab,kw OR 'extracorporeal dialysis':ti,ab,kw

#8 'hypoxia-inducible factor prolyl hydroxylase inhibitors':ti,ab,kw OR 'hif phis':ti,ab,kw OR roxadustat:ti,ab,kw OR 'fg 4592':ti,ab,kw

#9 'crossover procedure':de OR 'double-blind procedure':de OR 'randomized controlled trial':de OR 'single-blind procedure':de OR (random* OR factorial* OR crossover* OR cross NEXT/1 over* OR placebo* OR doubl* NEAR/1 blind* OR singl* NEAR/1 blind* OR assign* OR allocat* OR volunteer*):de,ab,ti

#10 #1 OR #2 OR #3

#11 #4 OR #5

#12 #6 OR #7

#13 #8 AND #9 AND #10 AND #11 AND #12

CNKI:

TKA=Roxadustat * anemia * (hemodialysis + Dialysis)

Wanfang Database:

Title/key words: Roxadustat AND anemia AND (hemodialysis OR Dialysis)

CQ VIP:

Title/key words: Roxadustat AND anemia AND (hemodialysis OR Dialysis)

SinoMed Database:

(" Roxat "[title] AND " Anemia"[title] AND (" Hemodialysis "[title] OR "Renal Dialysis"[title] OR "Extracorporeal dialysis" ([Chinese title])) AND (clinical trial [publication type] OR randomized controlled trial [publication type] OR meta-analysis [publication type] OR multi-center study [publication type]) AND human [feature word]
